# Peripheral regional anaesthesia and outcomes: a narrative review of the literature from 2013 to 2023

**DOI:** 10.1016/j.bja.2023.10.013

**Published:** 2023-11-11

**Authors:** Manouk Admiraal, Peter Marhofer, Philip M. Hopkins, Markus W. Hollmann

**Affiliations:** 1 Department of Anaesthesiology, Amsterdam University Medical Center, Amsterdam, The Netherlands; 2 Department of Anaesthesia, General Intensive Care Medicine and Pain Medicine, Medical University of Vienna, Vienna, Austria; 3 Leeds Institute of Medical Research, University of Leeds, Leeds, UK

**Keywords:** anaesthesia, conduction block, data analysis, nerve block, peripheral nerves, regional anaesthesia

## Abstract

The use of peripheral regional anaesthesia continues to increase, yet the evidence supporting its use and impact on relevant outcomes often lacks scientific rigour, especially when considering the use of specific blocks for a particular surgical indication. In this narrative review, we consider the relevant literature in a 10-yr period from 2013. We performed a literature search (MEDLINE and EMBASE) for articles reporting randomised controlled trials and other comparative trials of peripheral regional anaesthetic blocks *vs* systemic analgesia in adult patients undergoing surgery. We evaluated measures of effective treatment and complications. A total of 128 studies met our inclusion criteria. There remains variability in the technical conduct of blocks and the outcomes used to evaluate them. There is a considerable body of evidence to support the use of interscalene blocks for shoulder surgery. Saphenous nerve (motor-sparing) blocks provide satisfactory analgesia after knee surgery and are preferred to femoral nerve blocks which are associated with falls when patients are mobilised early as part of enhanced recovery programmes. There are additional surgical indications where the efficacy of cervical plexus, intercostal nerve, and ilioinguinal/iliohypogastric nerve blocks have been demonstrated. In the past 10 yr, there has been a consolidation of the evidence indicating benefit of peripheral nerve blocks for specific indications. There remains great scope for rigorous, multicentre, randomised controlled trials of many peripheral nerve blocks. These would benefit from an agreed set of patient-centred outcomes.

**Editor's key points**

• A previous review of publications on peripheral regional anaesthesia published from 2003 to 2013 concluded that most techniques were beneficial and permanent complications were rare.
• This review addresses the next decade of evidence up to 2023, and compares the results to the results of the previous decade.
• The spectrum of clinical indications for purely sensory blocks is increasing, whereas some techniques are declining in importance.
• A lack of anatomical correlates exists for a number of techniques remains.

The use and importance of peripheral regional anaesthesia continues to increase. New findings in anatomy, pharmacodynamics, and pharmacokinetics have brought the practice of regional nerve blocks to the realm of personalised precision medicine. Unfortunately, only a small number of publications report good quality science, which (together with the heterogeneity of publications) is the main reason why most systematic reviews and meta-analyses can conclude only that more evidence is required to inform daily clinical practice.

In 2015, we published a narrative review article where the outcomes of peripheral regional anaesthesia techniques were analysed based on the relevant literature over a 10-yr period from 2003 to 2013.^1^ We were able to determine that the majority of peripheral regional blocks showed benefits for patients and that permanent complications were rare. We also showed which regional techniques are less useful for particular indications.

Many studies have been published since then, providing new findings and evidence regarding peripheral regional anaesthesia. Therefore, it is worthwhile to re-investigate the literature and to summarise relevant outcome data in a manner that will guide clinical practice and enable researchers to identify priorities for future investigations.

## Methods

### Search and selection

We conducted a narrative review by performing a systematic literature search from October 4, 2013 to February 16, 2023. MEDLINE and EMBASE (Ovid) were used to retrieve RCTs and other comparative studies that investigated the most commonly used peripheral nerve blocks. The search strategy was developed by a clinical librarian with particular experience in literature searches. A detailed description of the search is available in Supplementary material A. Using Rayyan software (Rayyan Systems Inc., Cambridge, MA, USA), two reviewers independently screened the titles and abstracts of the retrieved articles for eligibility. Any disagreements were resolved by a third reviewer. Thereafter, full-text articles were obtained and research articles published solely in the English language were further assessed.

### Study inclusion criteria

Participants: Patients aged 18 yr and older who were undergoing surgery.

Interventions: The most commonly used peripheral nerve block techniques for surgery on the upper extremity, lower extremity, and trunk.

Comparator: conventional therapy (systemic analgesia), placebo, or sham nerve blocks.

Outcomes: Effectiveness measures, such as block success rate, pain scores, amount of analgesic and/or anaesthetics needed, and opioid-related side effects; long-term outcomes, such as the incidence of chronic postsurgical pain and long-term functional recovery; patient satisfaction; short-term functional recovery; admission time; and complications, such as falls as a result of muscle weakness, paraesthesia, vocal cord paralysis, and subcutaneous emphysema.

### Study exclusion criteria

Studies comparing peripheral nerve block with neuraxial analgesia or local infiltration anaesthesia and studies investigating the combination of nerve blocks with fascial plane blocks were excluded. We also excluded animal studies, research on children, technical reports, dose-finding studies, and studies comparing different local anaesthetics.

## Results

**Fig 1:**
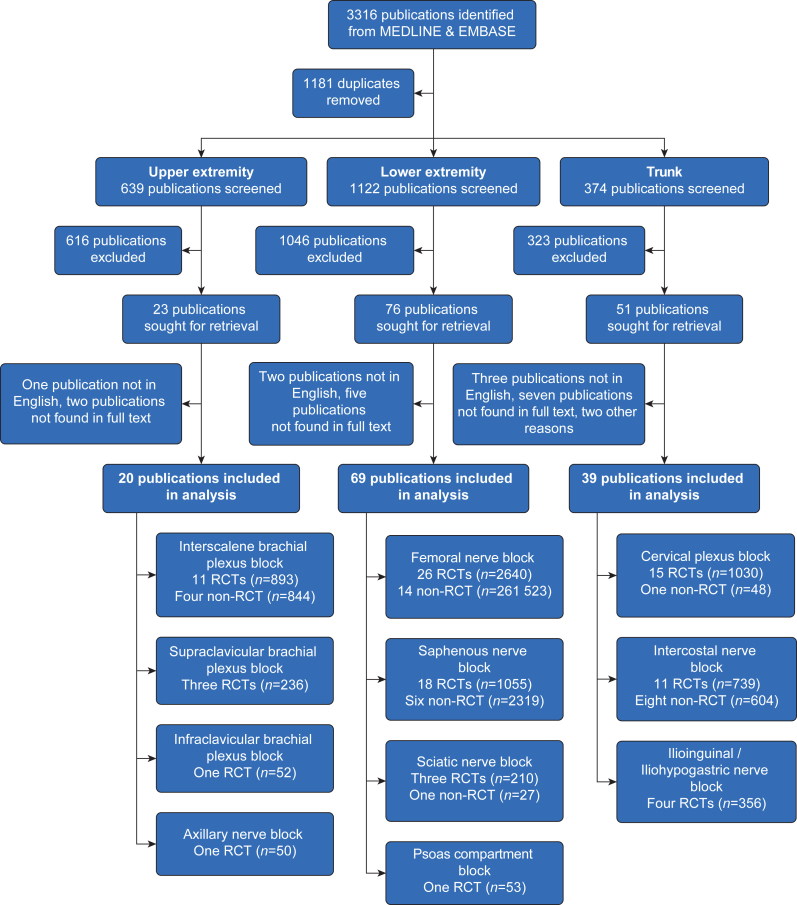
Flow diagram for inclusion and exclusion of studies.

The initial search on MEDLINE and EMBASE databases identified 3316 articles. After eliminating duplicates, 2135 studies were assessed for eligibility, with 128 studies ultimately included in this review. Figure 1 presents the flow diagram depicting the selection and exclusion process for the studies. Table 1 illustrates relevant differences between the respective narrative reviews of the past two decades (2003–13 and 2013–23).

**Table 1:** Relevant differences between outcome data for peripheral regional anaesthetic techniques during the last 20 yr (2003–23). TKA, total knee arthroplasty.

Regional anaesthetic technique	Summary of outcome between October 4 2003 and October 3, 2013	Summary of outcome between October 4, 2013 and February 16, 2023	Comparison between outcome data
Interscalene brachial plexus block	17 RCTs, 11 non-RCTs Adequate pain therapy, relatively high incidence of complications (associated with large volumes of local anaesthetics), comparisons with subacromial infiltration	11 RCTs, four non-RCTs Still well published, adequate pain therapy, less incidence of complications (because of more experience with ultrasound), one study (indication: mastectomy) outside the anatomical supply area	Slight decrease in scientific publications, sufficient pain therapy, decrease of reported complications
Supraclavicular brachial plexus block	One RCT, six non-RCTs Limited data, high incidence of phrenic nerve block, complications increase with increasing doses of local anaesthetics	Three RCTs, zero non-RCTs Still not well published, data in the field of forearm fracture show a high conversion rate to general anaesthesia	Still limited data, present data indicate an unreliable analgesic effect, possible surgical indications (e.g. elbow surgery) still not investigated in comparative RCTs
Infraclavicular brachial plexus block	One RCT, five non-RCTs Adequate pain therapy, shorter discharge times as compared with general anaesthesia, vascular puncture and high volumes of local anaesthetic are risks	One RCT, zero non-RCTs Adequate pain therapy with low rate of complications	Decrease in publications, adequate pain therapy, low rate of complications
Axillary approach to the brachial plexus	Two RCTs, nine non-RCTs Outcome data are insufficient, short-term management is improved, permanent nerve damage is described	No publications	No adequate studies in this field were published during the past 10 yr.
Axillary nerve block	No publications	One RCT, zero non-RCT Insufficient for shoulder surgery	Only one RCT during the last 20 yr, the technique is insufficient for shoulder surgery
Femoral nerve block	42 RCTs, five non-RCTs Large number of studies, sufficient pain relief after knee surgery (better as wound or intra-articular infiltration), chronic postsurgical pain not affected, insufficient pain therapy after hip fracture	26 RCTs, 14 non-RCTs Many studies in the field of hip surgery (questionable clinical indication), sufficient technique for knee surgery, because of reported postoperative falls, more peripheral techniques for knee surgery should be considered	Still many publications in the field of hip surgery without an anatomical correlate, sufficient for knee surgery, but more peripheral techniques should be considered because of reported postoperative falls
Saphenous nerve block	Eight RCTs, one non-RCT Effective pain therapy after TKA and knee arthroscopy, no motor block	18 RCTs, six non-RCTs Adequate pain therapy and improved functional recovery after knee surgery, better effect after TKA than after anterior cruciate ligament reconstruction	The increase in publications confirm the trend towards motor-sparing and more peripheral nerve block techniques.
Sciatic nerve block	23 RCTs, four non-RCTs Effective pain therapy, improved patient satisfaction	Three RCTs, one non-RCT Sufficient pain therapy	The significant decrease of publications indicate the trend to motor-sparing regional anaesthetic techniques, if performed, sciatic block is sufficient.
Psoas compartment block	Five RCTs, one non-RCT Sufficient pain therapy for hip surgery, equal to epidural anaesthesia, high incidence of epidural spread of local anaesthetic	One RCT, zero non-RCT Surgeon-performed, effective for hip surgery, no information regarding pharmacodynamic values	The significant decrease in publications indicate the trend to more peripheral regional anaesthetic techniques, anaesthesiologists do not study this technique anymore.
Cervical plexus block	12 RCTs, zero non-RCT High patient satisfaction for thyroid and carotid surgery	15 RCTs, one non-RCT Still well published with a broader spectrum of indications including ear surgery, only minor complications reported	The numbers of publications are similar between the decades, the spectrum of indications increase, further indications could be included in RCT (e.g. clavicle surgery)
Intercostal nerve block	Nine RCTs, one non-RCT Good pain therapy for rib fractures, thoracic surgery, and laparoscopic procedures, postsurgical pulmonary function similar to thoracic epidural anaesthesia	11 RCTs, eight non-RCTs Additional procedures are now included, surgical performance of block appears as less efficient	The number of publications increased with a broader spectrum of indications. The block should be performed by anaesthesiologists.
Ilioinguinal/iliohypogastric nerve block	Six RCTs, two non-RCTs Earlier hospital discharge after hernia repair compared with pure general anaesthesia	Four RCTs, zero non-RCT Caesarean section is now included in the spectrum of indications, safe technique	The number of indications increased (Caesarean section), can be considered as safe technique

### Upper extremity blocks

Supplementary material B provides an overview of included articles involving upper extremity blocks.

#### Interscalene brachial plexus block

##### Eleven RCTs (*n*=893) and four non-RCTs (*n*=844)

The interscalene plexus block remains the most extensively studied regional anaesthetic technique of the upper extremity despite a decline in the number of publications. Shoulder surgery continues to be the most researched indication (13 studies),^2^, ^3^, ^4^, ^5^, ^6^, ^7^, ^8^, ^9^, ^10^, ^11^, ^12^, ^13^, ^14^ with one study each reporting the use of interscalene plexus block for mastectomy,^15^ and upper extremity fracture surgery.^16^

Ultrasound guidance was used in the majority of studies (*n*=13),^2^, ^3^, ^4^, ^5^^,^^7^, ^8^, ^9^, ^10^, ^11^, ^12^, ^13^^,^^16^ whereas two studies used nerve stimulation to guide needle placement.^14^^,^^15^ Two studies investigated the effectiveness of continuous infusion of local anaesthetic through a perineural catheter.^10^^,^^11^ Five studies did not define primary outcome measures,^4^^,^^5^^,^^11^^,^^12^^,^^16^ and among those that did, opioid consumption was the most commonly studied outcome variable (five studies).^3^^,^^6^^,^^9^^,^^13^^,^^15^ Two RCTs reported a total of three failed blocks out of 495 in RCTs performed.^5^^,^^6^ Four studies described a total of five complications in 401 patients,^2^^,^^6^^,^^10^^,^^15^ with four further studies reporting no complications in 184 patients^4^^,^^7^^,^^14^^,^^16^ resulting in an incidence of less than 1% (Supplementary material B). Recurrent laryngeal nerve paralysis was observed in three patients, two of which resolved the day after surgery and one that resolved after 3–6 months.^2^^,^^6^ One patient experienced breathing difficulties that resolved within 2 h.^10^

##### Shoulder surgery

Interscalene brachial plexus block was described in nine RCTs (*n*=733) and four non-RCTs (*n*=844) for patients undergoing shoulder surgery, mainly arthroscopic. After surgery, six RCTs (*n*=393),^5^^,^^7^^,^^8^^,^^10^^,^^11^^,^^14^ and three non-RCTs (*n*=693),^2^^,^^9^^,^^13^ reported reduced pain scores in the groups receiving interscalene block, whereas one retrospective cohort study reported a similar pain experience (*n*=151),^3^ and one RCT initially reported less pain, but after 72 h more pain (*n*=66).^12^ With the exception of two studies (*n*=225),^13^^,^^14^ the majority of investigations on postoperative analgesia revealed a reduced need for postoperative medication (four RCTs [*n*=372],^5^^,^^6^^,^^10^^,^^11^ and three non-RCTs [*n*=702]).^2^^,^^3^^,^^9^ During hospitalisation, functional recovery was enhanced in one RCT (*n*=120),^6^ and similar between groups in another RCT (*n*=96).^11^ One study reported improved shoulder function 6 weeks after surgery, but this improvement was not sustained after 6 months (*n*=85).^5^ One RCT found a shorter PACU stay (*n*=120),^6^ with shorter hospitalisation time (*n*=71).^10^

##### Mastectomy

Patients who underwent modified radical mastectomy, which involves the removal of the entire breast and some axillary lymph nodes, showed a significant decrease in opioid usage during the initial 24-h period when administered interscalene plexus block compared with those who received general anaesthesia without a block.^15^ The rationale for this finding is, however, obscure.

##### Summary statement

Interscalene brachial plexus block is nowadays performed with ultrasound guidance and is mainly described for shoulder surgery, where most of the publications indicate adequate pain therapy with subsequent effects of reduced length of stay in the PACU and shorter hospitalisation time. The one publication, where mastectomy served as an indication for interscalene brachial plexus block, is an example of the misinterpretation of the anatomical basis of regional anaesthesia.

Although previous publications between 2003 and 2013 described a relatively high occurrence of complications, recent studies report a lower incidence of complication rates, which might be explained by greater use of, and experience with, the ultrasound-guided technique.

#### Supraclavicular brachial plexus block

##### Three RCTs (*n*=236)

No studies comparing the use of perioperative supraclavicular block with general anaesthesia in surgical patients met the inclusion criteria for our previous review,^1^ although a single RCT with a sample size of 12 patients reported the use of supraclavicular block in an emergency department. Our current literature search has also found a limited degree of research activity in this area since then. Two studies examined the effectiveness of supraclavicular block for pain management during endovascular treatment of dysfunctional arteriovenous fistulae,^17^^,^^18^ whereas another study focused on patients undergoing radial fracture fixation.^19^

##### Endovascular treatment of dysfunctional arteriovenous fistulae

Two RCTs (*n*=148) demonstrated that supraclavicular block provided higher patient satisfaction and lower pain scores immediately after the procedure compared with sedo-analgesia.^17^^,^^18^ In addition, in the sedo-analgesia group, five patients experienced severe oxygen desaturation perioperatively, whereas no complications were reported in the supraclavicular block group.

##### Radial fracture fixation

A study involving 88 patients found that those who received a supraclavicular block had lower opioid usage on the first postoperative day and experienced less pain than those who received general anaesthesia alone.^19^ However, 16% of patients who received the block required unplanned general anaesthesia because their block was insufficient. Furthermore, three patients allocated to the general anaesthesia group required a rescue block after surgery because of pain. Long-term outcome measurements, such as functionality and patient-reported outcome measurement, were similar between groups.

##### Summary statement

As in the previous review, few studies were published regarding supraclavicular block. The supraclavicular block appears superior to sedo-analgesia in patients undergoing endovascular treatment. Only one study investigated the effectiveness of supraclavicular block during radial fracture fixation with a high conversion rate to general anaesthesia in patients allocated to receive supraclavicular block. Based on the limited data currently available, no evidence supports the use of supraclavicular block for this particular surgery. Possible useful indications for this regional anaesthetic technique, such as its use for elbow surgery, have still not been studied.

#### Infraclavicular brachial plexus block

##### One RCT (*n*=52)

Only one RCT with 52 patients undergoing radial fracture fixation was conducted between 2013 and 2023.^20^ In this study, only one patient required conversion to general anaesthesia because of an insufficient block (=3.8% failure rate). Ultrasound-guided infraclavicular block was found to be more effective in terms of patient satisfaction, pain scores, and reduction of nausea when compared with conventional analgesia. However, no differences were found in functional outcomes at 3 and 6 months after the procedure.

##### Summary statement

Despite the paucity of publications from 2003 to 2013, only one RCT has been published in the subsequent period. Nevertheless, infraclavicular brachial plexus block appears to be a regional anaesthetic technique with low failure and complication rates.

#### Axillary nerve block

##### One RCT (*n*=50)

Limited research is available on axillary nerve blocks with only one RCT identified. This study found a slight reduction in postoperative pain scores in patients who received an axillary nerve block during arthroscopic shoulder surgery.^21^ However, only 70.3% of the axillary nerve blocks were successful and 42% of the patients with a successful axillary nerve block required an interscalene rescue block because of inadequate pain control. No significant difference was found in opioid consumption between groups.

##### Summary statement

Axillary nerve block, unsurprisingly, appears to be an insufficient regional anaesthetic technique for shoulder surgery.

### Lower extremity blocks

Supplementary material C shows an overview of included articles.

#### Femoral nerve block

##### Twenty-six RCTs (*n*=2640) and 14 non-RCTs (*n*=261 523)

Femoral nerve block is still the most frequently investigated nerve block of the lower extremity. Up until 2013, it was mainly described for use with knee surgery (28 studies)^1^ and in subsequent years there have been 13 RCTs (*n*=1457).^22^, ^23^, ^24^, ^25^, ^26^, ^27^, ^28^, ^29^, ^30^, ^31^, ^32^, ^33^, ^34^ More recently, there has been an increase in the emphasis of scientific investigations involving hip surgery, whereas only three studies were published from 2003 to 2013. In the current period, femoral nerve block is described for hip surgery (total hip arthroplasty or hip arthroscopy),^35^, ^36^, ^37^, ^38^, ^39^, ^40^, ^41^, ^42^, ^43^, ^44^, ^45^, ^46^, ^47^ femoral fracture,^48^, ^49^, ^50^ knee surgery (total knee arthroplasty, anterior cruciate ligament reconstruction),^22^, ^23^, ^24^, ^25^, ^26^, ^27^, ^28^, ^29^, ^30^, ^31^, ^32^, ^33^, ^34^^,^^51^, ^52^, ^53^, ^54^, ^55^, ^56^, ^57^ and tibial fracture/osteotomy.^58^^,^^59^ Endovenous laser ablation is also described as an indication for femoral nerve block.^60^^,^^61^

The majority of femoral nerve blocks used ultrasound guidance (22 studies),^23^^,^^26^^,^^28^, ^29^, ^30^, ^31^, ^32^, ^33^^,^^35^, ^36^, ^37^^,^^39^^,^^42^^,^^47^, ^48^, ^49^^,^^54^^,^^56^^,^^57^^,^^59^, ^60^, ^61^ but 10 studies used nerve stimulation,^22^^,^^27^^,^^34^^,^^40^^,^^41^^,^^44^, ^45^, ^46^^,^^50^^,^^52^ and eight studies did not report the nerve identification technique or were not clear in their description.^24^^,^^25^^,^^38^^,^^43^^,^^51^^,^^53^^,^^55^^,^^58^

The outcome variables reported for evaluation of femoral block were heterogeneous, with 18 studies reporting a primary outcome.^22^^,^^24^^,^^26^, ^27^, ^28^^,^^31^^,^^32^^,^^37^^,^^38^^,^^41^, ^42^, ^43^, ^44^^,^^47^^,^^48^^,^^57^^,^^58^^,^^61^ A positive effect on pain intensity was mainly detected when ultrasound was used (16 studies),^23^^,^^26^^,^^28^^,^^31^, ^32^, ^33^^,^^35^^,^^36^^,^^39^^,^^47^^,^^48^^,^^56^^,^^57^^,^^59^, ^60^, ^61^ but five studies where nerve stimulation guidance was used also reported a positive effect,^34^^,^^41^^,^^45^^,^^46^^,^^50^ as did two studies with an unknown needle guidance technique.^24^^,^^55^ However, one study showed an increased pain intensity in patients with femoral nerve block compared with patients without.^29^ In contrast, 16 studies did not investigate or show any significant benefits on pain intensity after femoral nerve block.^22^^,^^25^^,^^27^^,^^30^^,^^33^^,^^37^^,^^38^^,^^40^^,^^42^, ^43^, ^44^, ^45^^,^^49^^,^^51^, ^52^, ^53^, ^54^^,^^58^

In 26 RCTs examining a total of 1425 femoral nerve blocks, 13 falls were reported in patients with a nerve block (incidence 0.9%).^22^^,^^27^^,^^28^^,^^47^

##### Hip surgery

Seven RCTs (*n*=812)^37^^,^^38^^,^^41^^,^^44^, ^45^, ^46^, ^47^ and six non-RCTs (*n*=1915)^35^^,^^36^^,^^39^^,^^40^^,^^42^^,^^43^ investigated femoral nerve block for hip surgery. An increase in the number of investigations of femoral nerve block for hip surgery can be observed between 2013 and 2023.

Four RCTs (*n*=448)^41^^,^^45^, ^46^, ^47^ and two non-RCTs (*n*=361)^35^^,^^39^ reported reduced analgesia demand when femoral nerve block was used for hip surgery, with the remaining studies demonstrating similar consumption (*n*=849),^36^, ^37^, ^38^^,^^40^^,^^45^ or not investigating analgesic effects (*n*=1069).^42^^,^^43^ The heterogenic results are in accordance with the anatomical rationale for using femoral nerve block for hip surgery, where only the anterior hip capsule is innervated by articular branches of the femoral nerve.^62^

Three RCTs (*n*=178)^37^^,^^38^^,^^47^ and one non-RCT (*n*=96)^39^ investigated the use of preoperative femoral nerve block. Although the intraoperative effect of preoperative femoral nerve block seems to be insignificant, all available studies report sufficient postoperative pain therapy. Nevertheless, it needs to be highlighted that one RCT^47^ reported 22% of postoperative falls.

Other investigated outcomes were postoperative nausea and vomiting (PONV; six studies, where two showed a positive effect in the femoral nerve group).^35^^,^^36^ In addition, one study reported more adverse drug effects without further explanation.^44^ Length of hospital stay was reported in four studies, without any differences between groups,^38^^,^^40^^,^^42^^,^^44^ length of PACU stay in two studies (where stays were shorter in the femoral nerve block group,^41^ or similar between groups^39^), postoperative mobilisation in two studies (with femoral nerve block showed a faster mobilisation),^42^^,^^43^ and postoperative delirium (similar between groups in two studies).^35^^,^^44^ One study reported a higher incidence of falls (six patients).^47^

##### Femoral fracture surgery

Femoral nerve block for femoral fracture repair is described in three RCTs (*n*=262). Two of these studies (*n*=171) reported a positive effect on perioperative pain,^48^^,^^50^ whereas one study (*n*=91) did not find any pain-related effects.^49^ No effects on other outcome variables were detected.

##### Knee surgery

According to the number of reported cases, total knee arthroplasty seems to be the major indication for femoral nerve block. Eight RCTs (*n*=1223),^22^^,^^24^^,^^25^^,^^28^^,^^31^, ^32^, ^33^, ^34^ and five non-RCTs (*n*=259 245)^51^^,^^53^^,^^55^, ^56^, ^57^ report femoral nerve block for total knee arthroplasty.

All RCTs except two^22^^,^^33^ reported lower postoperative pain scores and less perioperative systemic analgesia requirements when femoral nerve block was used for total knee arthroplasty. The quality of two large retrospective studies is low, without descriptions of block performance, postoperative systemic analgesic demand, etc.^51^^,^^53^

The use of femoral nerve block for anterior cruciate ligament repair is reported in five RCTs (*n*=212)^23^^,^^26^^,^^27^^,^^29^^,^^30^ and two non-RCTs (*n*=303).^52^^,^^54^ Two RCTs showed a positive analgesic effect of femoral nerve block,^23^^,^^26^ and three studies did not show advantages in terms of pain therapy.^27^^,^^29^^,^^30^ In one of these studies, a landmark-based regional technique was performed,^27^ and one study with a negative effect compared the regional block with a continuous morphine infusion, where more nausea was detected.^29^ The two non-RCTs (*n*=303), where femoral nerve block was used for anterior cruciate ligament repair, did not investigate postoperative pain therapy.^52^^,^^54^

No differences in functional recovery were reported.^54^ Two falls were detected after anterior cruciate ligament repair under femoral nerve block.^27^

##### Tibial surgery

Two RCTs (*n*=83) describe femoral nerve block for surgery of the (proximal) tibia. Both studies did not show significant additional analgesic effects of femoral nerve block.^58^^,^^59^

##### Summary statement

The increase in publications where femoral nerve block is used for hip surgery is a good example of when the choice of regional anaesthesia technique is not based on sound anatomical considerations. All in all, the effect of femoral nerve block for hip surgery is questionable. In contrast, all types of knee surgery are an appropriate indication for femoral nerve block. However, the association of falls after femoral nerve block suggests that the alternative use of a more distal and mainly sensory block (saphenous nerve/adductor canal) should be considered (see below).

#### Saphenous nerve block

##### Eighteen RCTs (*n*=1055) and six non-RCTs (*n*=2319)

An analysis of the literature reveals a notable increase in the number of publications that have investigated the use of the saphenous nerve block between 2013 and 2023. Between 2003 and 2013, only nine publications were identified, whereas 24 studies were published in the subsequent decade (2013–23). The primary indication for the saphenous nerve block was knee surgery (23 studies),^63^, ^64^, ^65^, ^66^, ^67^, ^68^, ^69^, ^70^, ^71^, ^72^, ^73^, ^74^, ^75^, ^76^, ^77^, ^78^, ^79^, ^80^, ^81^, ^82^, ^83^, ^84^, ^85^ with one study examining tibial osteotomy.^86^

The majority of studies used ultrasound guidance (*n*=22) for the saphenous nerve block.^63^, ^64^, ^65^^,^^67^, ^68^, ^69^, ^70^, ^71^, ^72^, ^73^^,^^75^^,^^77^, ^78^, ^79^, ^80^, ^81^, ^82^, ^83^, ^84^, ^85^, ^86^ In one study the block was performed by the surgeon,^66^ whereas two others were unclear about the technique used to identify the nerve.^74^^,^^76^ Pain scores were the primary outcome in most studies (*n*=10), but the methods of assessment varied significantly across studies, with some reporting peak pain, others average pain, and some pain at rest or during exercise; the timing of assessments also differed. From studies with a pre-specified primary outcome, six demonstrated a reduction in pain in patients who received the saphenous nerve block.^63^^,^^64^^,^^72^^,^^73^^,^^77^^,^^86^

The perioperative anaesthetic and analgesic procedures in the respective studies were heterogeneous. Besides the saphenous nerve block, six studies used general anaesthesia only,^67^^,^^68^^,^^72^^,^^73^^,^^83^^,^^86^ four used spinal anaesthesia only,^65^^,^^76^^,^^80^^,^^85^ six combined spinal anaesthesia with local infiltration analgesia,^63^^,^^64^^,^^70^^,^^71^^,^^77^^,^^79^ one combined general anaesthesia with local infiltration analgesia,^75^ two used epidural analgesia,^78^^,^^81^ and in five studies various methods were used or the anaesthetic method was unclear.^66^^,^^69^^,^^74^^,^^82^^,^^84^

None of the studies reported complications related to nerve block procedures.

##### Knee surgery

Saphenous nerve block has been examined in a total of 10 RCTs (*n*=612)^64^^,^^70^^,^^71^^,^^73^^,^^77^, ^78^, ^79^, ^80^, ^81^^,^^85^ and six non-RCTs (*n*=2319)^63^^,^^69^^,^^74^^,^^82^, ^83^, ^84^ in patients undergoing knee arthroplasty, three RCTs (*n*=139) for anterior cruciate ligament reconstruction,^67^^,^^75^^,^^76^ and four RCTs (*n*=269) for arthroscopic knee surgery.^65^^,^^66^^,^^68^^,^^72^

Of these studies, 12 showed a positive analgesic effect of saphenous nerve block compared with conventional systemic analgesia in patients undergoing total knee arthroplasty,^63^^,^^64^^,^^71^^,^^73^^,^^74^^,^^77^, ^78^, ^79^^,^^81^^,^^83^, ^84^, ^85^ but one RCT showed an increase in pain measured at 12 and 24 h after surgery,^80^ and three studies did not demonstrate any advantages in terms of pain therapy.^69^^,^^70^^,^^82^

One study did not find a difference between any of the investigated outcomes in patients undergoing anterior cruciate ligament reconstruction with a saphenous nerve block,^67^ but two studies observed minor improvements in pain management.^75^^,^^76^

Early in-hospital functional recovery was either improved (four studies with a total of 785 patients)^64^^,^^77^^,^^84^^,^^85^ or found to be similar between groups (five studies with 585 patients).^63^^,^^71^^,^^78^^,^^79^^,^^82^ One study found similar long-term functional recovery, evaluated using range of motion.^78^ Although one study reported less PONV,^80^ most studies (*n*=1123) demonstrated a similar incidence of adverse effects.^64^^,^^65^^,^^67^^,^^68^^,^^70^^,^^73^^,^^75^^,^^79^^,^^83^ Additionally, increased patient satisfaction was demonstrated in one study (*n*=40),^79^ whereas similar satisfaction was found in five studies (*n*=925).^70^^,^^75^^,^^77^^,^^83^^,^^85^

##### Summary statement

Saphenous nerve block appears to be a safe and effective regional anaesthetic technique for knee surgery. Saphenous nerve block improves pain management in patients undergoing knee surgery and may be also early functional recovery after total knee arthroplasty.

#### Sciatic nerve block

##### Three RCTs (*n*=210) and one non-RCT (*n*=27)

We detected a decline in the number of publications related to sciatic nerve block, with only four articles published since 2013 in comparison with 27 articles published from 2003 to 2013. One possible explanation for this observation could be the increased emphasis on early postoperative mobilisation. In patients who received a sciatic nerve block for pain treatment during endovascular treatment below the knee, a complete motor block was observed in three (10%) patients.^87^

Previously, research on sciatic nerve blocks was mainly focused on *knee surgery*. However, a wide range of procedures has been examined in recent years, including endovenous laser ablation,^87^ foot and ankle surgery,^88^^,^^89^ and traumatic lower limb amputation.^90^

Ultrasound guidance was used in three out of four of the studies, and this was associated with good outcomes regarding perioperative effects.^87^^,^^88^^,^^90^ One study used nerve stimulation for continuous sciatic nerve block.^89^

##### Lower limb amputation

In patients undergoing traumatic limb amputation, single-shot sciatic nerve block did not result in a reduction in the incidence nor the severity of chronic phantom limb pain compared with placebo.^90^

##### Summary statement

In our prior review,^1^ we included 27 studies that investigated the effectiveness of a sciatic nerve block, often combined with a femoral nerve block. A noticeable trend has emerged towards motor-sparing nerve blocks for lower extremity surgery, resulting in a decrease in popularity of the traditional sciatic block. However, despite this decline in the number of studies, positive perioperative analgesic effects were reported in patients who underwent a sciatic nerve block.

#### Psoas compartment block

##### One RCT (*n*=53)

The lack of interest in the psoas compartment block, also called lumbar plexus block, may be because of its relatively high risks of complications. The main complication was epidural diffusion with a varying incidence of 3–27% in a previously published meta-analysis.^91^ Although it produces good analgesic effects, the incidence of epidural spread of local anaesthetics was higher compared with other forms of regional anaesthesia.

Only one RCT has investigated the effectiveness of surgeon-performed psoas compartment block in patients undergoing total hip arthroplasty. The authors reported lower pain scores (without clarifying the scoring method) and a longer time to first analgesia in favour of patients with the block, although no measure of dispersion of the data was reported.^92^

##### Summary statement

Despite its ability to provide sufficient pain relief, the popularity of psoas compartment block is decreasing. Safer and more peripheral regional anaesthesia alternatives have emerged.

### Trunk blocks

Supplementary material D shows an overview of included articles.

#### Cervical plexus block

##### Fifteen RCTs (*n*=1030) and one non-RCT (*n*=48)

Until 2013, cervical plexus block was mainly studied in relation to thyroid surgery (nine studies). Although thyroid surgery still remains the most extensively studied indication for cervical plexus block with nine RCTs (*n*=612),^93^, ^94^, ^95^, ^96^, ^97^, ^98^, ^99^, ^100^, ^101^ other types of surgery, including middle ear surgery,^102^^,^^103^ parathyroidectomy,^104^^,^^105^ carotid endarterectomy,^106^ cervical discectomy and fusion,^107^ and craniotomy^108^ have also been described.

The majority of cervical plexus nerve blocks were performed using ultrasound guidance (nine studies),^96^^,^^98^^,^^99^^,^^101^, ^102^, ^103^, ^104^, ^105^^,^^108^ whereas six studies used a landmark-based technique,^93^^,^^95^^,^^97^^,^^100^^,^^106^^,^^107^ and one study did not report the specific method of needle guidance.^94^

Ten studies (*n*=646) specified a primary outcome measure. Out of these 10 studies, improvement in the quality of recovery, as measured by the Quality of Recovery (QoR) questionnaire, was observed in three studies.^101^^,^^105^^,^^107^ Perioperative opioid consumption was reduced in four studies,^96^^,^^100^^,^^103^^,^^108^ pain levels reduced in two studies,^102^^,^^104^ and a shorter time to discharge was observed in one study,^106^ all in favour of patients who received a cervical plexus block compared with those who received systemic analgesia only.

Hoarseness was observed in 10 patients who received cervical plexus nerve block,^96^^,^^98^^,^^107^ with symptoms resolved within 6–12 h after surgery. In addition, two patients experienced postoperative subcutaneous emphysema,^100^ which decreased within 24 h. All of these minor complications (*n*=10) were observed in patients undergoing thyroid surgery. No major complications were reported. The overall incidence of minor complications in the 526 cervical plexus nerve blocks performed in RCTs was 1.9%.

##### Thyroid surgery

Nine RCTs (*n*=612)^93^, ^94^, ^95^, ^96^, ^97^, ^98^, ^99^, ^100^, ^101^ investigated the use of cervical plexus nerve block for pain control during thyroidectomy. All of these studies reported positive effects on pain intensity or opioid consumption. Other outcome measurements that were investigated included patient satisfaction (*n*=144), which showed improvement in one study^101^ and no significant difference in another study,^99^ quality of recovery, and length of PACU stay (*n*=72), which showed improvement and shorter stays, respectively.^101^ The incidence of PONV was reduced in two studies,^95^^,^^101^ with a similar incidence between groups in three studies.^93^^,^^99^^,^^108^ Because of the small sample sizes and low incidence of PONV, it was not possible to draw conclusions regarding differences between the groups.

##### Parathyroidectomy

Two studies (*n*=130) evaluated the effects of cervical plexus nerve block for pain control after parathyroidectomy. Both studies reported a reduction in pain and decreased use of perioperative analgesia.^104^^,^^105^ In addition, one study found a statistically significant improvement in quality of recovery.^105^

##### Tympanic and mastoid surgery

A single RCT demonstrated reduced intraoperative opioid use and a lower incidence of PONV in patients who underwent tympanomastoid surgery with cervical plexus block.^103^ In patients who underwent mastoidectomy, lower levels of postoperative pain were observed in those who received cervical plexus block than in those who received general anaesthesia alone.^102^

##### Summary statement

The indications for cervical plexus block were broader than those in our previous narrative review.^1^ Cervical plexus block provides adequate perioperative pain management for a variety of surgical indications with only minor complications (all resolving within 24 h). From an anatomical perspective, surgery of the clavicle could also be included in the spectrum of indications, but no studies have been published to investigate the efficacy of cervical plexus block for surgery of the clavicle.

#### Intercostal nerve block

##### Eleven RCTs (*n*=739) and eight non-RCTs (*n*=604)

In the period before 2013, studies of intercostal nerve block were conducted to investigate pain relief in patients with rib fractures and patients undergoing laparoscopic or thoracic surgery. Since 2013, (video-assisted) thoracic surgery seems to be the main indication for studies of intercostal nerve block (eight RCTs^109^, ^110^, ^111^, ^112^, ^113^, ^114^, ^115^, ^116^ and six non-RCTs).^117^, ^118^, ^119^, ^120^, ^121^, ^122^ However, other indications include percutaneous nephrolithotomy,^123^^,^^124^ breast surgrery,^125^^,^^126^ and minimally invasive mitral valve surgery.^127^

We found that various techniques of intercostal nerve block have been reported. Most blocks were performed via thoracoscopic visualisation (*n*=4),^115^^,^^118^, ^119^, ^120^ or under direct vision by the surgeon (*n*=5).^111^^,^^112^^,^^121^^,^^122^^,^^126^ In four studies, the block was performed using ultrasound guidance,^110^^,^^113^^,^^116^^,^^124^ two studies used landmark techniques,^109^^,^^125^ one study used landmark in combination with fluoroscopy,^123^ and three studies did not report a needle guidance technique.^114^^,^^117^^,^^127^

Out of 14 studies that used intercostal nerve block for thoracic surgery, 13 reported a reduction in pain, a decrease in the consumption of opioid/non-opioid analgesics, or both.^109^, ^110^, ^111^^,^^113^, ^114^, ^115^, ^116^, ^117^, ^118^, ^119^, ^120^, ^121^, ^122^ Furthermore, all studies conducted for indications other than thoracic surgery demonstrated, although sometimes minor, improvement in pain management.^123^, ^124^, ^125^, ^126^, ^127^ None of the studies reported complications associated with the block, except for one case of spontaneously resolving pneumothorax, which was likely related to the placement of a port-a-cath.^126^

##### Thoracic surgery

The most studied procedure in relation to intercostal nerve blocks was video-assisted thoracic surgery (VATS) with two RCTs (*n*=155)^109^^,^^115^ and four non-RCTs (*n*=258).^118^, ^119^, ^120^^,^^122^ In addition, four studies (*n*=305) investigated the use of intercostal nerve blocks in open thoracotomy^112^^,^^114^^,^^116^^,^^121^ and four studies (*n*=814) in sternotomy.^110^^,^^111^^,^^113^^,^^117^

For VATS, all reviewed studies showed a decrease in opioid consumption and lower pain scores in patients who received intercostal nerve blocks (*n*=413).^109^^,^^115^^,^^118^^,^^119^^,^^122^^,^^125^ Additionally, a reduced hospital length of stay was demonstrated in a subset of studies (*n*=178).^118^^,^^122^ Positive outcomes were also observed in all studies that examined patients undergoing sternotomy compared with patients without an intercostal nerve block.^110^^,^^111^^,^^113^^,^^117^

Five studies reported on thoracic surgery and the incidence of PONV. Two RCTs (*n*=171) reported less PONV in patients with intercostal nerve block,^113^^,^^116^ whereas three RCTs (*n*=196) demonstrated similar incidences.^109^^,^^110^^,^^115^ For patients with intercostal nerve block, a higher patient satisfaction score was demonstrated compared with patients without this therapy in two RCTs (*n*=136).^110^^,^^115^

##### Percutaneous nephrolithotomy

Two RCTs (*n*=103) reported reduced analgesic requirements when intercostal nerve block was performed for percutaneous nephrolithotomy. Moreover, greater satisfaction was demonstrated in one study,^124^ and improved health-related quality of life was shown in another.^123^

##### Breast surgery

Two non-RCTs involving a total of 176 patients compared intercostal nerve block with conventional systemic analgesia in patients undergoing breast surgery. The results showed a significant reduction in pain^125^ and a shorter length of stay, which may lead to anticipated cost reductions ranging from $1500 to $3000 per patient.^126^ These projected cost savings were estimated by using a patient's hospital bill as a proxy for expenses. However, when considering the perspective of a third-party payer, the projected costs using Medicare reimbursement were identical across both groups, as Medicare reimbursement did not vary based on extended hospital stays.

##### Summary statement

VATS was the most frequently studied procedure and showed improved pain management in patients who received intercostal nerve block compared with those who did not. In open thoracotomy, intercostal nerve blocks performed by the surgeon do not seem to offer benefits. The spectrum of indications seems to increase and recently investigated procedures such as percutaneous nephrolithotomy and breast surgery showed improved outcomes in patients who received intercostal nerve blocks.

#### Ilioinguinal and iliohypogastric nerve block

##### Four RCTs (*n*=356)

The ilioinguinal and iliohypogastric nerve block has primarily been studied in the context of Caesarean section in two RCTs,^128^^,^^129^ whereas one RCT mentioned the ilioinguinal nerve block only.^130^ Additionally, one study investigated the effectiveness of pain management in patients undergoing surgery for cervical cancer.^131^

Two RCTs (*n*=230) used the landmark technique for nerve identification,^129^^,^^130^ whereas another two RCTs (*n*=126) used ultrasound guidance.^128^^,^^131^

All studies reported positive outcomes of the ilioinguinal and iliohypogastric nerve block on postoperative pain scores and analgesic requirements.

One case of haematoma at the catheter site was reported which resolved spontaneously.^129^ Notably, for Caesarean delivery, the incidence of chronic postsurgical pain appears to be lower in patients receiving ilioinguinal/iliohypogastric nerve block than in those receiving spinal anaesthesia alone.^128^

##### Summary statement

In our previous review, ilioinguinal/iliohypogastric nerve block was predominantly studied in the context of hernia repair. Subsequently, this block has been evaluated for its effectiveness in Caesarean delivery, yielding positive outcomes in terms of pain reduction and analgesia requirements, without relevant complications.

## Discussion

We designed a follow-up narrative review regarding the literature covering peripheral regional anaesthetic techniques from the past 10 yr. Our previous narrative review of this field, analysing scientific publications from 2003 to 2013, included 142 RCTs and non-RCTs, whereas this review detected 128 RCTs and non-RCTs applicable for analyses. Thus, we analysed a similar number of scientific publications in each of the past two decades.

We assume that the number of scientific publications serves as a surrogate measure for the interest in specific topics; the dramatically increasing number of publications in the field of fascial plane blocks is an example of this. Despite this burgeoning interest in fascial plane blocks, we believe that the use of specific regional anaesthetic techniques having robust anatomical bases will provide the best and most consistent results. Therefore, continued scientific development in the field of peripheral nerve block is essential. The format of a narrative review represents the clinical picture and the practical impact of regional anaesthesia in an adequate manner. The drawback of most systematic reviews and meta-analyses is the final statement that the current literature is not sufficient to draw definitive conclusions in the particular field of interest, which is mainly because of the heterogenous study concepts and outcome measures.^132^ With the present narrative review, we provide clinically relevant information regarding developments in peripheral regional anaesthesia during the last decade and a practical overview of the current literature which can be used to guide daily clinical practice. According to our findings in this narrative review, future clinical studies in relevant fields can be designed.

The scientific publications during the last decade indicate an ongoing trend in increased use of peripheral regional anaesthetic techniques, but this is block-specific. Psoas compartment block, for example, was rarely investigated and this perhaps highlights the growing emphasis on motor-sparing lower limb blocks now the benefits of early mobilisation and enhanced recovery programmes are appreciated. Indeed, we found that femoral block is associated with postoperative falls, whereas saphenous nerve block preserves motor function and provides satisfactory analgesia after knee surgery.

The increasing number of indications for peripheral nerve blocks to provide regional anaesthesia of the trunk—cervical plexus block, intercostal nerve block, ilioinguinal/iliohypogastric nerve block in our review—is the second example for the increasing use of pure sensory regional blocks. The difference between these blocks and fascial plane blocks is the presence of an anatomical correlate, enabling a precision ultrasound-guided technique. This is likely to be associated with greater consistency of outcome and reduced local anaesthetic dose requirements.

We were disappointed to find further examples of scientific publications where regional anaesthetic techniques were used without comprehensible anatomical correlates. Interscalene brachial plexus block for mastectomy is one example of this. In particular, the large number of femoral nerve block studies for hip surgery during the last decade is surprising, considering the fact that insufficient pain therapy after hip fracture was reported in our previous narrative review. In contrast, some promising indications for peripheral regional anaesthetic techniques have not been adequately investigated. Surgery of the clavicle under cervical plexus block and elbow surgery under supraclavicular brachial plexus block are two examples.

Although some techniques are not published on anymore because they are (becoming) obsolete, such as the psoas compartment block, there may be reasons why more popular blocks are under-represented in our literature search. Clinical studies of the axillary approach to the brachial plexus, for example, feature prominently in PubMed (more than 60 studies in the past 10 yr) but almost all of these did not meet our inclusion criteria. This might be because clinicians and researchers consider the scope, efficacy, and risks of the technique to be resolved.

According to the findings between 2003 and 2023, some recommendations can be made: (1) interscalene brachial plexus block is an efficient technique for shoulder surgery with a low rate of complications (when correctly performed); (2) femoral nerve block is not useful for hip surgery and should not be used for knee surgery whenever the concept of fast-track surgery is used because of reported falls, despite its clinical efficacy; (3) saphenous nerve block is the preferred regional anaesthetic technique for total knee arthroplasty; (4) psoas compartment and axillary brachial plexus block have lost importance and are now irrelevant; and (5) the number of useful clinical indications for cervical plexus block, intercostal nerve block, and ilioinguinal/iliohypogastric nerve block is increasing.

Despite a large body of literature, the overall scientific quality of the research in the field of peripheral regional anaesthesia is limited. Despite specific publications regarding the definition of outcomes in peripheral regional anaesthesia,^133^ there is still no definite consensus regarding the most relevant outcome measures. The Core Outcome Measures for Perioperative and Anaesthetic Care (COMPAC) initiative, where mortality, perioperative complications, resource use, and short-/long-term recovery are suggested as useful variables, also revealed the widespread inconsistency in outcome reporting.^134^

Therefore, comparison of publications is still difficult. From the technical perspective, the main outcome variables could be defined as duration of surgical sensory block and the time to first opioid analgesic request. One of the challenges in this context is that many regional techniques are performed together with opioid-based general anaesthesia. Complications of regional blocks can serve as a further technical outcome measure, but as nerve damage or systemic toxicity is extremely rare, these are probably best addressed through registry studies or large (national) audits. Alternatively, we recommend that the field of regional anaesthesia research embraces the concept of patient-centred outcomes, such as QoR-15 reported in a few of the identified studies. Use of such outcomes is to be encouraged in order to provide a more holistic picture of the merits of regional anaesthetic techniques.

Another drawback of the research we reviewed is that it consists almost entirely of relatively small single-centre studies. Large multicentre studies, especially RCTs, would improve the generalisability of research findings and enable the use of more rigorous statistical significance criteria, thereby reducing the risk of false-positive inference.

In conclusion, we noticed some progress in the quality of scientific publications over the last decade (2013–2023). Clear recommendations can be made for some specific regional anaesthetic techniques. The main finding is a trend towards more peripheral and motor-sparing regional anaesthetic techniques. Despite a large body of scientific literature, some techniques need more attention in future clinical studies. We noticed a lack of scientific publications in the field of periclavicular brachial plexus blocks and for particular indications of cervical plexus block. Conversely, further studies in the field of femoral nerve block and hip surgery are not necessary.

## Authors’ contributions

Study design and concept: all authors.

Manuscript preparation: all authors.

## Acknowledgements

We thank Faridi S. van Etten-Jamaludin, clinical librarian, University Medical Center, Amsterdam, The Netherlands, for valuable assistance in the electronic literature search.

## Declaration of interest

PMH is editor-in-chief of *BJA Open*. All other authors declare no conflicts of interest.

## Funding

Departmental sources.
