# Supplementary material for: Peripheral regional anaesthesia and outcomes: a narrative review of the literature from 2013 to 2023
Source: Br J Anaesth. 2023 Nov 11;132(5):1082–96. doi: 10.1016/j.bja.2023.10.013 (PMC11103102; doi:10.1016/j.bja.2023.10.013)
Supplement: Multimedia component 1 [file mmc1.docx]

**Upper extremity**

| Databases: |  |  |
| --- | --- | --- |
| PubMed, Embase (Ovid) | Before deduplication | After deduplication |
| Total | 996 | 639 |

**PubMed search 17-2-2023:** 414 hits

(((interscalene nerve block*[tiab] OR interscalene plexus block*[tiab] OR interscalene brachial plexus block*[tiab] OR supraclavicular nerve block*[tiab] OR supraclavicular plexus block*[tiab] OR supraclavicular brachial plexus block*[tiab] OR infraclavicular nerve block*[tiab] OR infraclavicular plexus block*[tiab] OR infraclavicular brachial plexus block*[tiab] OR axillary nerve block*[tiab] OR axillary plexus block*[tiab] OR axillary brachial plexus block*[tiab]) AND ("Randomized Controlled Trial" [Publication Type] OR "Comparative Study" [Publication Type] OR random*[tiab] OR comparative stud*[tiab])) NOT ("Systematic Review" [Publication Type] OR "Meta-Analysis" [Publication Type] OR "Review" [Publication Type] OR "Guideline" [Publication Type] OR systematic review[ti] OR meta-analysis[ti])) AND (("2013/10/03"[Date - Publication] : "2023/02/17"[Date - Publication]))

**EMBASE (OVID):**

Database(s): Embase Classic+Embase 1947 to 2023 February 16
Search Strategy:

| **#** | **Searches** | **Results** |
| --- | --- | --- |
| 1 | (interscalene nerve block* or interscalene plexus block* or interscalene brachial plexus block* or supraclavicular nerve block* or supraclavicular plexus block or supraclavicular brachial plexus block* or infraclavicular nerve block* or infraclavicular plexus block* or infraclavicular brachial plexus block* or axillary nerve block* or axillary plexus block* or axillary brachial plexus block*).ti,ab,kf. | 2740 |
| 2 | exp controlled study/ or comparative study/ or (random* or comparative stud*).ti,ab. | 11379352 |
| 3 | 1 and 2 | 1316 |
| 4 | exp "review"/ or exp "systematic review"/ or exp meta analysis/ or exp practice guideline/ or (systematic review or meta-analysis).ti. | 3809686 |
| 5 | 3 not 4 | 1232 |
| 6 | limit 5 to conference abstract status | 243 |
| 7 | 5 not 6 | 989 |
| 8 | limit 7 to yr="2013 -Current" | 582 |

**Lower extremity**

| Databases: |  |  |
| --- | --- | --- |
| PubMed, Embase (Ovid) | Before deduplication | After deduplication |
| Total | 1735 | 1122 |

**PubMed search 17-2-2023:** 723 hits

((("Sciatic Nerve"[Mesh] OR "Femoral Nerve"[Mesh]) AND "Nerve Block"[Mesh]) OR (sciatic block*[tiab] OR sciatic nerve block*[tiab] OR femoral block*[tiab] OR femoral nerve block*[tiab] OR saphenous block*[tiab] OR saphenous nerve block*[tiab] OR adductor canal block*[tiab] OR psoas compartment block*[tiab])

AND

("Randomized Controlled Trial" [Publication Type] OR "Comparative Study" [Publication Type] OR random*[tiab] OR comparative stud*[tiab]))

NOT

("Systematic Review" [Publication Type] OR "Meta-Analysis" [Publication Type] OR "Review" [Publication Type] OR "Guideline" [Publication Type] OR systematic review[ti] OR meta-analysis[ti])

AND

("2013/10/03"[Date - Publication] : "2023/02/17"[Date - Publication])

**EMBASE (OVID)**

Database(s): Embase Classic+Embase 1947 to 2023 February 16
Search Strategy:

| **#** | **Searches** | **Results** |
| --- | --- | --- |
| 1 | (sciatic block* or sciatic nerve block* or femoral block* or femoral nerve block* or saphenous block* or saphenous nerve block* or adductor canal block* or psoas compartment block*).ti,ab,kf. | 4346 |
| 2 | (*"sciatic nerve"/ or *"femoral nerve"/ or *saphenous nerve/) and "nerve block"/ | 1883 |
| 3 | 1 or 2 | 4746 |
| 4 | exp controlled study/ or comparative study/ or (random* or comparative stud*).ti,ab. | 11379352 |
| 5 | 3 and 4 | 2449 |
| 6 | exp "review"/ or exp "systematic review"/ or exp meta analysis/ or exp practice guideline/ or (systematic review or meta-analysis).ti. | 3809686 |
| 7 | 5 not 6 | 2191 |
| 8 | limit 7 to conference abstract status | 523 |
| 9 | 7 not 8 | 1668 |
| 10 | limit 9 to yr="2013 -Current" | 1003 |

**Trunk:**

| Databases: |  |  |
| --- | --- | --- |
| PubMed, Embase (Ovid) | Before deduplication | After deduplication |
| Total | 585 | 374 |

**PubMed search 17-2-2023:** 514 hits

((cervical plexus block*[tiab] OR intercostal block*[tiab] OR intercostal blockade*[tiab] OR intercostal nerve block*[tiab] OR ilioinguinal block*[tiab] OR ilioinguinal nerve block*[tiab] OR iliohypogastric block*[tiab] OR iliohypogastric nerve block*[tiab])

AND

("Randomized Controlled Trial" [Publication Type] OR "Comparative Study" [Publication Type] OR random*[tiab] OR comparative stud*[tiab]))

NOT

("Systematic Review" [Publication Type] OR "Meta-Analysis" [Publication Type] OR "Review" [Publication Type] OR "Guideline" [Publication Type] OR systematic review[ti] OR meta-analysis[ti])

AND ("2013/10/03"[Date - Publication] : "2023/02/15"[Date - Publication])

**EMBASE (OVID):**

Database(s): **Embase Classic+Embase**1947 to 2023 February 16
Search Strategy:

| **#** | **Searches** | **Results** |
| --- | --- | --- |
| 1 | (cervical plexus block* or intercostal block* or intercostal blockade* or intercostal nerve block* or ilioinguinal block* or ilioinguinal nerve block* or iliohypogastric block* or iliohypogastric nerve block*).ti,ab,kf. | 2160 |
| 2 | exp controlled study/ or comparative study/ or (random* or comparative stud*).ti,ab. | 11537019 |
| 3 | 1 and 2 | 856 |
| 4 | exp "review"/ or exp "systematic review"/ or exp meta analysis/ or exp practice guideline/ or (systematic review or meta-analysis).ti. | 3858687 |
| 5 | 3 not 4 | 777 |
| 6 | limit 5 to conference abstract status | 132 |
| 7 | 5 not 6 | 645 |
|  | limit 7 to yr="2013 -Current" | 367 |
