# Supplementary material for: Peripheral regional anaesthesia and outcomes: a narrative review of the literature from 2013 to 2023
Source: Br J Anaesth. 2023 Nov 11;132(5):1082–96. doi: 10.1016/j.bja.2023.10.013 (PMC11103102; doi:10.1016/j.bja.2023.10.013)
Supplement: Multimedia component 2 [file mmc2.docx]

**Interscalene**

| **Author** | **Design** | **N** | **Surgery** | **Technique** | **Anaesthetics** | **Primary outcome** | **Result primary outcome** | **Success rate** | **Pain** | **Analgesia + anaesthetics** | **PONV or other side effects** | **Long-term outcomes** | **Other (quality of recovery, satisfaction, functional recovery, hospital stay)** | **Complications** |
| --- | --- | --- | --- | --- | --- | --- | --- | --- | --- | --- | --- | --- | --- | --- |
| Bosco et al.^1^ | Retrospective | 425 | Arthroscopic shoulder | US - single shot  (20-30 mL of 0.5% ropivacaine:2% lidocaine as a 1:1 mixture) | *4 groups:*  - 124 IBPB  -72 IBPB*pre* + GA - 52 IBPB*post* + GA  - GA | Time to discharge from PACU | IBPB shortest time (mean (SD) 71 (40) min. vs. 89 (50) vs. 108 (48) vs. 111 (57), p < 0.001) |  | Incidence moderate to severe pain higher in GA only group,  at PACU arrival (30 (47%) patients vs. 26 (36%) vs. 26 (50%) vs. 117 (67%), p < 0.001) and at PACU discharge (16 (13%) patients vs. 21 (29%) vs. 10 (19%) vs. 68 (39%), p < 0.001 ) | Proportion requiring postoperative analgesia higher in GA only group (50 (38%) patients vs. 44 (61%) vs. 34 (66%) vs. 157 (89%).  ** No p-values is mentioned* | Lower incidence N/V (19 (15%) patients vs. 9 (13%) vs. 14 (27%) vs. 50 (28%), p = 0.008) |  |  | 1 vocal cord paralysis, resolved with voice therapy in 3-6 months |
| Chen et al.^2^ | Retrospective | 151 | Elective shoulder | US - single shot  (20 mL of 0.25% levobupivacaine) | 103 IBPB + GA vs. GA | Total amount of IV PCA-analgesics (3.3 mcg fentanyl + 1 mg ketorolac /ml) | - IPBP less analgesics in first 24 hr (mean (SD) 58 (23) ml vs. 87 (34), p < 000.1) and in first 48 hr (mean 115 (41) ml vs. 184 (45), p < 000.1) |  | No dif. in VAS at 24, 48 hr |  | - No dif. in N/V.  - Lower incidence of dizziness 2% vs. 15%, p = 0.005) |  |  |  |
| Choi et al.^3^ | RCT | 152 | Arthroscopic rotator cuff repair | US + NS - single shot  (10 mL of 0.25% ropivacaine with 200 mcg of epinephrine) | 73 IBPB + GA vs. GA |  |  |  |  | Less frequent IO administration analgesia (mean (SD) 0.19 (0.46) times vs. 0.53 (0.86), p = 0.003). |  |  |  | None related to block |
| Gurger et al.^4^ | RCT | 85 | Arthroscopic rotator cuff repair | US + NS - single shot  (30 ml of 0.25% bupivacaine) | 42 IBPB + GA vs. GA |  |  | 2 failed blocks | Lower VAS at 1hr (mean (SD) 4.7 (1.1) vs. 7.3 (1.4), at 6 hr (3.7 (1.2) vs. 5.4 (1.2)), at 12 hr (3.3 (1.1) vs. 4.8 (1.1)), at 24 hr (2.9 (0.8) vs. 3.9 (1), all p < 0.001). | - More patients did not need postoperative analgesics (20 (48%) patients vs. 6 (14%), p < 0.001)  - Less patients required IV tramadol after surgery (8 (19%) patients vs. 27 (63%), p < 0.001) |  | Improved shoulder function at 6 weeks (Constant score mean (SD) 67 (9) vs. 59 (8), p <0.001). No dif. at 6 months.  ** Higher score (= better function)* | No dif. in LOS |  |
| Janssen et al.^5^ | RCT | 83 | Shoulder arthroscopy | NS - single shot  (30 ml bupivacaine 0.25 %) | 41 IBPB + GA vs. GA | Incidence of arterial hypotension requiring treatment | n.a. | 100%, no rescue block required | Lower NRS on POD 0 (mean (SD) 2.7 (2.6) vs. 4.1 (2.6), p = 0.02). No dif. POD 1 en 2. | - No dif. in (non-)opioid consumption after surgery  - No dif. in opioid consumption during surgery  - No dif. in propofol requirement during surgery | No dif. PONV | No dif. in satisfaction |  | None related to block |
| Kaya et al.^6^ | RCT | 60 | Modified radical mastectomy | NS - single shot  (10 ml mepivacaine 1 % and 20 ml ropivacaine 0.375 %) | 30 IBPB + GA vs. GA | Total 24-hr IV morphine consumption | Less IV morphine (median [range] 5 [0-40] mg vs. 22 [6-48] mg, p = 0.001)) |  | Lower VAS at 0, 1, 2, 4, 6, 12 hr (p < 0.007)  ** No actual VAS scores are mentioned* |  | - Lower incidence nausea (14 (47%) patients vs. 25 (83%), p = 0.03).  - No dif. in other side-effects |  |  | 2 patients signs of Horner’s syndrome |
| Lehmann et al.^7^ | RCT | 120 | Arthroscopic shoulder | US – single shot  (20 ml of 0.5% levobupivacaine with 100 µg of epinephrine) | *3 groups*  - 40 IBPB  - 40 GA + IBPB  - GA | Opioid consumption at POD 0 | Less patients used opioids (10 patients vs. 10 vs. 25, p = 00037) | IBPB group:  - 1 failed block resulting in GA  - 20 patients received additional sedation |  | - No dif. in analgesia requirements POD 1 to POD 4  and  - Less IO sufentanil in IBPB + GA vs. GA (median [range] 25 [20-50] mcg vs. 40 [20-70], p <0.001).  - Less propofol in IBPB + GA vs. GA (median [range] 150 [100-250] mg vs. 200 [120-330] mg, p = 0.0037) | - Less N/V 2 vs. 4 vs. 10, p = 0.0015 |  | - Satisfaction improved (median [range] 10 [4-10] vs. 9 [3-10] vs. 9 [0-10], p = 0.0134)- Shorter PACU time (median [range] 35 [5-106] min. vs. 58 [11-220] vs.93 [5-182], p < 0.001)  - Faster ambulation time (median [range] 85 [11-1156] min. vs. 195 [85-500] vs. 272 [75-1231], p < 0.001) | 2 patients with IBPB developed paresis recurrent laryngeal nerve (full recovery the day after surgery) |
| Lim et al.^8^ | RCT | 40 | Arthroscopic shoulder | US + single shot  (2 mg/kg of 0.75% [ropivacaine](https://www.sciencedirect.com/topics/medicine-and-dentistry/ropivacaine) and 3 mg/kg of 2% lidocaine with a 30-mL max) | 20 IBPB + GA vs. GA | Body temperature at 120 minutes after induction | N.a. |  | More patients with a lower NRS in PACU at 15 min (NRS 0, 12 patients vs. 0, NRS 1-3, 8 vs. 13, NRS 4-6, 0 vs. 7, NRS 7-10, 0 vs. 0, p < 0.001) |  |  |  | Lower concentration desflurane at 15 minutes (mean (SD) 5 (0.8%) vs. 5.8 (0.4), p = 0.001) and 120 min (3.4% (0.4) vs. 7.1 (0.9), p < 0.001) | None related to block |
| Meija-Terrazas et al.^9^ | RCT | 20 | Arthroscopic shoulder | US + NS – single shot  (20 mL of bupivacaine 0.5% with epinephrine 1:200,000) | 10 IBPB vs.  GA | Cytokines release | N.a. |  | Lower VAS immediately after surgery (median [IQR] 0 [0-0] vs. 4 [2-4], p <0.001). No dif. at 12 and 24 hr |  |  |  |  |  |
| Olofsson et al.^10^ | Before/after + matching | 126 | Fixation clavicle fracture | US + continuous vs. single shot  (20 mL 0.5% ropivacaine) | 50 IBPB + GA vs. GA | Total IV morphine consumption at 2 hr upon departure PACU | Less morphine (mean dif. 8.3 IV MME, 95% CI 6.5 to 10.0, p < 0.001)  *After matching:* Lower mean dif. 9.9 IV MME (95% CI 6.7-13, p < 0.001) | 100% success | *After matching:*  Lower NRS in rest at 2 hr (mean dif. 1.7, 95% CI 0.8 to 2.5, p < 0.001).  No dif. in NRS in rest at 24 hr | *After matching:*  - Lower IO sufentanil (mean dif. 28 mcg, 95% CI 24-33, p < 0.001).  - Less morphine 24 hr (mean dif. 9.9 IV MME, 95% CI 6.7 to 13.0, p < 0.001) | *After matching:*  No dif. in PONV within 24 hr |  |  |  |
| Salviz et al.^11^ | RCT | 71 | Arthroscopic rotator cuff repair | US + NS – single shot  (10–20 mL of 0.125–0.25% levobupivacaine) | *3 groups*  20 cIBPB + sedation vs. 23 sIBPB + sedation vs. GA | Difference in proportion with NRS < 4 on POD 7 | Lower proportion NRS < 4 (74% vs 17% vs 42%, p < 0.05). | 1 catheter was accidentally removed | No difference in proportions NRS on POD 1, 2, 3 | Use of ≥ 1 doses of narcotics lower in cIBPB than other groups on POD 1 (60% patients vs. 96% vs. 90%, p <0.01) and POD 2 (65% s. 96% s. 95%, p < 0.01). No dif. on POD 3. | No dif. in PONV |  | - Shorter PACU for both IBPB vs GA (mean (SD) 20 minutes (31) vs. 30 (42) vs. 165 (118), p < 0.001)  - Shorter hospitalization for both IBPB vs GA (mean (SD) 94 (55) minutes vs. 115 (109) vs. 302 (249), p < 0.05) | One patient in cIBPB had transient difficulty breathing on arrival to the PACU, resolved within 2 hours. |
| Shin et al.^12^ | RCT | 96 | Arthroscopic rotator cuff repair | US – continuous vs. single shot  (bolus 10 ml 0.2 % ropivacaine) | *3 groups*  31 cIBPB + GA vs.  32 sIBPB + GA vs. GA |  |  | 3 patients accidently pulled out catheter | Lower NRS cIBPB and sIBPB than GA at 1, 4, 6, 16, 21, 40 hr, p < 0.05). No dif. at PACU or 48 hr. ** No values mentioned* | Lower rate of taking supplementary opioids cIBPB and sIBPB than GA (5 (16.1%) patients vs. 6 (18.8%) vs. 14 (42.4%), p < 0.05) | No dif. in PONV or other side effects |  | No dif. in manual muscle test scoring at 1, 4, 6, 16, 21, 40 hr, 48 hr |  |
| Takahashi et al.^13^ | RCT | 66 | Arthroscopic rotator cuff repair | US – single shot  (0.75% ropivacaine 20 ml and 0.5% bupivacaine 10 ml) | 43 IBPB + GA vs. GA |  |  |  | - Lower VAS for night pain first 24 hr (mean (SD) 7.1 (2.9) vs. 8.6 (1.6), p = 0.017) and higher at 72 hr (mean 4.6 (2.5) vs. 3.3 (2.5), p < 0.024). No dif. at 48 hr.  - No dif. at rest or movement 24, 48, 72 hr |  |  |  |  |  |
| Yuan et al.^14^ | RCT | 100 | Upper extremity fractures + age > 70 years | US – single shot (25 mL mixing liquid of 0.375% [ropivacaine](https://www.sciencedirect.com/topics/medicine-and-dentistry/ropivacaine) and 1% lidocaine) | 50 IBPB + GA vs. GA |  |  |  | Lower incidence of incision pain (1 (2%) patient vs. 8 (16%), p = 0.014) | -Lower consumption propofol (mean (SD) 295 (69) mg vs. 337 (77), p = 0.004)  - Lower consumption isoflurane (mean (SD) 16 (3) mg vs. 23 (3), p < 0.001)  - Less use of additional analgesic drugs IO (3 (6%) vs. 14 (28%), p < 0.01. | No dif. in PONV |  | Faster recovery time (mean (SD) 117 (32) minutes vs. 133 (37), p = 0,02) | None related to block |
| Wu et al.^15^ | Retrospective | 142 | Shoulder arthroscopy | US – single shot  (15 mL 0.5% levobupivacaine and 5 mL 2% lidocaine) | 74 IBPB + GA vs. GA | IO morphine MME consumption | Lower (median [IQR] 0.07 [0.05 – 0.115] vs. 0.11 [0.078-0.1340 mg/kg/h, p < 0.001) |  | Lower VAS at PACU (median 0.5 [0-1] vs. 3 [3-4], p < 0.001) and at 24 hr after surgery during movement (median 2 [1-2] vs. 3 [3-4], p < 0.001) and rest (median 1 [0-1] vs. 3 [1-2], p < 0.001). | - No dif. in opioid consumption PACU and ward  - Lower sevoflurane consumption (median {IQR] 0.22 [0.18 – 0.27] vs. 0.24 [0.20 – 0.29] mL/kg/h, p = 0.001) | No dif. in PONV |  | - No dif. in satisfaction.  - No dif. in length of hospitalization |  |

**Abbreviations:** N = number of patients, PONV = postoperative nausea and vomiting, RCT = randomised controlled trial, US = ultrasound, IBPB = continuous interscalene brachial plexus block, GA = general anaesthesia, PACU = post anaesthesia care unit, N/V = nausea/vomiting, IV = intravenous, PCA = patient controlled analgesia, NS = nerve stimulation, VAS = visual analogue scale, LOS = length of stay, n.a. = not applicable, NRS = numeric rating scale, POD = postoperative day, MME = morphine equivalent, IO = intraoperative

**Supraclavicular**

| **Author** | **Design** | **N** | **Surgery** | **Technique** | **Anaesthetics** | **Primary outcome** | **Result primary outcome** | **Success rate** | **Pain** | **Analgesia + anaesthetics** | **PONV or other side effects** | **Long-term outcomes** | **Other (quality of recovery, satisfaction, functional recovery, hospital stay)** | **Complications** |
| --- | --- | --- | --- | --- | --- | --- | --- | --- | --- | --- | --- | --- | --- | --- |
| Gedikoglu et al.^16^ | RCT | 68 | Angioplasty dysfunctional arteriovenous access | US – single shot | SBPB vs. sedoanalgesia |  |  |  | Lower VAS score immediately after procedure (median [IQR} 0 [0-4] vs 6 [2-10], p < 0.001 |  |  |  | - Higher patient satisfaction (not satisfied 0 patients vs. 14 (41.2%) and completely satisfied 33 (97%) vs. 1 (3%), p < 0.001) – Higher surgeon satisfaction (p < 0.001) | None related to block procedure, however 5 severe oxygen desaturations occurred in sedoanalgesia group |
| Heo et al.^17^ | RCT | 80 | Angioplasty dysfunctional arteriovenous access | US – single shot | SBPB vs. local lidocaine + IV tramadol |  |  | 100% | Lower VAS immediately after procedure (mean (SD) 0.9 (1.9) vs. 6.4 (2.5), p < 0.001) |  |  |  | Higher patient satisfaction (mean (SD) 2.8 (0.5) vs. 2.1 (0.8), p < 0.001) | No dif. |
| Rundgren et al.^18^ | RCT | 88 | Radial fracture with plate fixation | US – single shot | SBPB vs. GA | Total oral opioid equivalent consumption 72 hr | No dif. | 7 converted to GA due to inadequate SBPB, 3 patients in GA had postop rescue block | Less pain at 0 hr (median [width IQR] 0 [0] vs. 6 [7], p < 0.001), 2 hr (0 [0] vs. 2 [3], p < 0.001), 24 hr (3 [3] vs. 2 [4], p < 0.001). No dif. at 48, 72 hr. | Lower oral opioid equivalent consumption POD 1 (median [range] 23 [0-75] mg vs. 35 [0-120], p < 0.001). No dif. POD 2 and 3. | No dif. | - No dif. in 6 months functional outcomes and EQ-5D or PRWE scores  - No dif. in pain scores at 2 weeks and 6 months. |  |  |

**Abbreviations:** N = number of patients, PONV = postoperative nausea and vomiting, RCT = randomised controlled trial, US = ultrasound, SBPB = supraclavicular brachial nerve block, VAS = visual analogue scale, IV = intravenous, GA = general anaesthesia, dif. = difference, POD = postoperative day, EQ-5D = EuroQoL-5 Dimension, PRWE = patients-related wrist-specific functioning

**Infraclavicular**

| **Author** | **Design** | **N** | **Surgery** | **Technique** | **Anaesthetics** | **Primary outcome** | **Result primary outcome** | **Success rate** | **Pain** | **Analgesia + anaesthetics** | **PONV or other side effects** | **Long-term outcomes** | **Other (quality of recovery, satisfaction, functional recovery, duration hospital stay)** | **Complications** |
| --- | --- | --- | --- | --- | --- | --- | --- | --- | --- | --- | --- | --- | --- | --- |
| Wong et al.^19^ | RCT | 52 | Radial fracture fixation | US – single shot | ICNB + sedation vs. GA | NRS with movement at 24 hr | Lower NRS (median 2 [2-4.2] vs. 6 [4-7.3], p = 0.001) | 1 converted to GA due to inadequate ICNB | - Lower NRS at rest in PACU (median [IQR] 0[0-0] vs. 5 [2-8]), 1 hr (0 [0-0] vs. 5 [5-8]), 2 hr (0 [0-0] vs. 5 [4-8], 24 hr (1.5 [0-3] vs. 4.5 [3-6], 48 hr (1 [0-2] vs. 4.5 [3-6]), all p ≤ 0.001.  - Lower NRS at movement in PACU (0[0-0] vs. 7.5 [3-9]), 1 hr (0 [0-0] vs. 6 [5-8]), 2 hr (0 [0-0] vs. 6.5 [4-8], 48 hr (2 [2-3] vs. 5 [4-7]), all p ≤ 0.001. | Lower IV morphine consumption PACU (median 0 [0-0] vs. 2.3 [0-3] mg, p < 0.001), no dif. in ward at 2/24/48 hr. | Less nausea (0% vs. 31%), less vomiting (0% vs. 19%, weakness in operated limb (8% vs. 31%, p = 0.035). No dif. in other side-effects | - No dif. in functional scores by PRWE and QuickDash, SF12, HADS at 3 and 6 months.  - No dif. in NRS at 3 and 6 months. | Higher patient satisfaction on POD 1 (p = 0.003) and at 3 months (p = 0.007) | None |

**Abbreviations:** N = number of patients, PONV = postoperative nausea and vomiting, RCT = randomised controlled trial, US = ultrasound, ICNB = infraclavicular nerve block, GA = general anaesthesia, NRS = numeric rating scale, PACU = post anaesthesia care unit, IV = intravenous, PRWE = patients-related wrist-specific functioning, SF = short-form, HADS = hospital anxiety and depression scale, POD = postoperative day.

**Axillary**

| **Auhor** | **Design** | **N** | **Surgery** | **Technique** | **Anaesthetics** | **Primary outcome** | **Result primary outcome** | **Success rate** | **Pain** | **Analgesia + anaesthetics** | **PONV or other side effects** | **Long-term outcomes** | **Other (quality of recovery, satisfaction, functional recovery, hospital stay)** | **Complications** |
| --- | --- | --- | --- | --- | --- | --- | --- | --- | --- | --- | --- | --- | --- | --- |
| Rothe et al.^20^ | RCT | 50 | Arthroscopic subacromial decompression | US - single shot | ANB + GA vs. placebo ANB + GA | IV morphine consumption 0-4 hr postop | No dif. | - 19/27 ANB successful  - 14 GA needed escape block (10 ANB vs 4 placebo). | Lower VAS 0-4 hr, measured as AUC (median [range] 135 [4-293] vs. 182 [15-383] mm, p = 0.03, lower at 8 hr (9 [0-26] vs. 20 [0-67] mm, p = 0.01) and 24 hr (0 [0-20] vs. 10 [0-45] mm, p = 0.04) | No dif. in oral morphine 4-24 hr | No dif. in PONV |  |  | None |

**Abbreviations:** N = number of patients, PONV = postoperative nausea and vomiting, RCT = randomised controlled trial, US = ultrasound, ANB = axillary nerve block, GA = general anaesthesia, VAS = visual analogue scale, AUC = area under the curve.

**References**

1. Bosco L, Zhou C, Murdoch JAC, Bicknell R, Hopman WM, Phelan R, et al. Pre- or postoperative interscalene block and/or general anesthesia for arthroscopic shoulder surgery: a retrospective observational study. Can J Anaesth. 2017,64(10):1048-58.

2. Chen HP, Shen SJ, Tsai HI, Kao SC, Yu HP. Effects of Interscalene Nerve Block for Postoperative Pain Management in Patients after Shoulder Surgery. Biomed Res Int. 2015,2015:902745.

3. Choi S, Kim T, Kwon YS, Kang H. Intra-operative effect of interscalene brachial plexus block to arthroscopic rotator cuff repair surgery. Int Orthop. 2019,43(9):2117-24.

4. Gurger M, Ozer AB. A comparison of continuous interscalene block versus general anesthesia alone on the functional outcomes of the patients undergoing arthroscopic rotator cuff repair. Eur J Orthop Surg Traumatol. 2019,29(8):1659-66.

5. Janssen H, Stosch R, Pöschl R, Büttner B, Bauer M, Hinz JM, et al. Blood pressure response to combined general anaesthesia/interscalene brachial plexus block for outpatient shoulder arthroscopy. BMC Anesthesiol. 2014,14:50.

6. Kaya M, Oguz G, Senel G, Kadiogullari N. Postoperative analgesia after modified radical mastectomy: The efficacy of interscalene brachial plexus block. Journal of Anesthesia. 2013,27:862-7.

7. Lehmann LJ, Loosen G, Weiss C, Schmittner MD. Interscalene plexus block versus general anaesthesia for shoulder surgery: a randomized controlled study. Eur J Orthop Surg Traumatol. 2015,25(2):255-61.

8. Lim SH, Lee W, Park J, Kim MH, Cho K, Lee JH, et al. Preoperative interscalene brachial plexus block aids in perioperative temperature management during arthroscopic shoulder surgery. Korean J Anesthesiol. 2016,69(4):362-7.

9. Mejia-Terrazas GE, Ruiz-Suarez M, Vadillo-Ortega F, Franco y Bourland RE, Lopez-Munoz E. Effect of interscalene nerve block on the inflammatory response in shoulder surgery: a randomized trial. Journal of Shoulder and Elbow Surgery. 2019,28:e291-e303.

10. Olofsson M, Taffe P, Kirkham KR, Vauclair F, Morin B, Albrecht E. Interscalene brachial plexus block for surgical repair of clavicle fracture: A matched case-controlled study. BMC Anesthesiology. 2020,20.

11. Salviz EA, Xu D, Frulla A, Kwofie K, Shastri U, Chen J, et al. Continuous interscalene block in patients having outpatient rotator cuff repair surgery: a prospective randomized trial. Anesth Analg. 2013,117(6):1485-92.

12. Shin SW, Byeon GJ, Yoon JU, Ok YM, Baek SH, Kim KH, et al. Effective analgesia with ultrasound-guided interscalene brachial plexus block for postoperative pain control after arthroscopic rotator cuff repair. J Anesth. 2014,28(1):64-9.

13. Takahashi R, Kajita Y, Harada Y, Iwahori Y, Deie M. Post-operative pain control following arthroscopic rotator cuff repair: Intravenous acetaminophen versus interscalene brachial plexus block, A prospective randomized study. J Orthop. 2021,25:6-9.

14. Yuan L, Tang W, Fu GQ, Wang J, Guo J, Chen WT. Combining interscalene brachial plexus block with intravenous-inhalation combined anesthesia for upper extremity fractures surgery: a randomized controlled trial. Int J Surg. 2014,12(12):1484-8.

15. Wu EB, Hsiao CC, Hung KC, Hung CT, Chen CC, Wu SC, et al. Opioid-Sparing Analgesic Effects from Interscalene Block Impact Anesthetic Management During Shoulder Arthroscopy: A Retrospective Observational Study. J Pain Res. 2023,16:119-28.

16. Gedikoglu M, Andic C, Guzelmansur I, Eker HE, Bolgen C. Comparison of Sedoanalgesia Versus Ultrasound-Guided Supraclavicular Brachial Plexus Block for the Prevention of the Pain During Endovascular Treatment of Dysfunctional Hemodialysis Fistulas. Cardiovasc Intervent Radiol. 2019,42(10):1391-7.

17. Heo S, Won JH, Kim J, Kim JY, Joe HB. Efficacy and Safety of Ultrasound-Guided Supraclavicular Brachial Plexus Block during Angioplasty of Dysfunctional Arteriovenous Access: A Prospective, Randomized Single-Center Clinical Trial. J Vasc Interv Radiol. 2020,31(2):236-41.

18. Rundgren J, Mellstrand Navarro C, Ponzer S, Regberg A, Serenius S, Enocson A. Regional or General Anesthesia in the Surgical Treatment of Distal Radial Fractures: A Randomized Clinical Trial. J Bone Joint Surg Am. 2019,101(13):1168-76.

19. Wong SS, Chan WS, Fang C, Chan CW, Lau TW, Leung F, et al. Infraclavicular nerve block reduces postoperative pain after distal radial fracture fixation: a randomized controlled trial. BMC Anesthesiol. 2020,20(1):130.

20. Rothe C, Lund J, Jenstrup MT, Steen-Hansen C, Lundstrøm LH, Andreasen AM, et al. A randomized controlled trial evaluating the impact of selective axillary nerve block after arthroscopic subacromial decompression. BMC Anesthesiol. 2020,20(1):33.
