# Supplementary material for: Peripheral regional anaesthesia and outcomes: a narrative review of the literature from 2013 to 2023
Source: Br J Anaesth. 2023 Nov 11;132(5):1082–96. doi: 10.1016/j.bja.2023.10.013 (PMC11103102; doi:10.1016/j.bja.2023.10.013)
Supplement: Multimedia component 3 [file mmc3.docx]

**Femoral nerve block**

| **Author** | **Design** | **N** | **Surgery** | **Technique** | **Anaesthetics** | **Primary outcome** | **Result primary outcome** | **Success rate** | **Pain** | **Analgesia** | **PONV or other side effects** | **Long-term outcomes** | **Other (quality of recovery, satisfaction, functional recovery, hospital stay)** | **Complications** |
| --- | --- | --- | --- | --- | --- | --- | --- | --- | --- | --- | --- | --- | --- | --- |
| Al Wahbi et al.^1^ | Prospective cohort | 60 | Endovenous laser ablation | US – single shot | 38 sFNB + TA vs. TA |  |  |  | Lower VAS during procedure (mean (SD) 1.1 (0.7) vs. 8.0 (0.7), p < 0.001) |  |  |  |  | None related to block |
| Angers et al.^2^ | RCT | 135 | TKA | NS – continuous vs. single shot | 3 groups:  45 cFNB + GA or spinal, vs. 45 sFNB + GA or spinal vs. 45 GA or spinal | Quadriceps strength recovery | - Reduced at 6 weeks (p < 0.01)  - Reduced at 6 and 12 months in closed chain contractions (p < 0.01 and p = 0.002). No dif. in open chain contractions |  | No dif. in VAS | No dif. in morphine consumption at 24 and 48 hours |  | - Lower ROM at 6 weeks (p = 0.046) and 12 months (p = 0.026) in both FNB groups. | - No dif. in LOS  - Worse (= lower) WOMAC score (p < 0.05) in both FNB groups  ** However, no values or time points mentioned* | 2 falls in FNB group in first week after surgery (extensor mechanism rupture and periprosthetic femoral fracture). |
| Arsoy et al.^3^ | Retrospective | 265 | Hip fractures in geriatric patients | US – continuous | 116 cFNC + ? vs. ? ** Unclear which method of anaesthesia* |  |  |  | Lower VAS POD 1 (mean (SD) 1.5 (1.6) vs. 3 (1.7), p < 0.001), and POD 2 (1.2 (1.5) vs. 2.6 (2.1), p < 0.001). | Less morphine consumption on POD 1 (mean (SD) 4.4 (5.8) MME vs. 7.2 (10.8), p < 0.001). No dif. POD 2 ** Unclear whether IV or oral MME* | - Less PONV (5.2% patients vs. 15.4%, p = 0.009).  - No dif. in delirium, urinary retention,  Ileus, respiratory depression |  | - More home discharge (14.6% vs. 6%, p = 0.023) | None related to block. |
| Arsoy et al.^4^ | Retrospective | 29 | THA in geriatric patients | US – continuous | 14 cFNC + GA or other vs. GA or other  ** Unclear what other is* |  |  |  | Lower VAS POD 1 (mean (SD) 1.6 (1.2) vs. 3.2 (1.6), p = 0.005), and POD 2 (1.8 (1.3) vs. 2.9 (1.4), p = 0.037) | No dif. in morphine consumption on POD 1 and 2. | Less opioid side effects (7.1% patients vs. 47%, p = 0.001) |  | - More home discharge (43% vs. 7%, p = 0.23).  - No dif. in LOS | None related to block. |
| Astur et al.^5^ | RCT | 30 | ACL reconstruction | US + NS – single shot | 16 sFNB + spinal + sedation vs. spinal + sedation |  |  |  | - Lower VAS at 6 hr (mean (SD) 0.4 (1) vs. 2.7 (2), p = 0.007).  - Higher VAS on POD 3 (4.6 (2.9) vs. 2.6 (2.5), p = 0.028), POD 6 (2.9 (2.9) vs. 1.9 (1.6), p = 0.026)  - No dif. at 12, 24, 28 hr, POD 4, 5, 7 | No dif. |  |  | - No dif. in knee flexion.  - No dif. in FIM scoring at 24 hour |  |
| Beaupre et al.^6^ | RCT | 73 | Hip fracture in patients ≥ 65 years | US – continuous until surgery, if surgery < 8 hr = single shot | 48 FNB + GA vs. GA. | VAS over time (POD 1 until 5) | No dif. |  |  | No dif. in total opioid use |  |  | No dif. number of patients who mobilized on POD 1 | None related to block. |
| Chan et al.^7^ | RCT | 200 | TKA | US and/ or NS – single vs. continuous | *Three groups:* 65 cFNB + spinal or GA vs. 69 sFNB + spinal or GA vs. spinal or GA. | Proportion VAS (> 4) during active knee flexion at 24 hr | Lower in both sFNB and cFNB compared to no-FNB (48 (69.6%) patients vs. 57 (86.4%) and 40 (61.5%) vs. 57 (86.4%), both p < 0.05). | In cFNB one patient failed catheter insertion due to technique difficulty and four were dislodged | - At rest lower VAS in cFNB vs. no-FNB at 6 hr (mean dif. -1.2, 95% CI -2.2 to -0.2, p = 0.018) and 24 hr (-0.7, 95% CI -1.2 to -0.2, p = 0.01).  - At movement lower VAS in both cFNB and sFNB vs. no-FNB at 24 hour (mean dif. -1.2, 95% CI -1.7 to -0.6, p < 0.001 and -0.6, 95% CI -1.1 to -0.01, p = 0.045). No dif. after 48 hr. | - Less opioid consumption in both cFNB and sFNB vs. no-PNB on POD 1 (mean (SD) 2.7 (2.6) vs. 21.2 (14.9) vs. 32.4 (26), p < 0.01) and on POD 2 (8.1 (7.1) vs. 32.5 (25.2) vs. 44.3 (36), p < 0.05) | - Less PONV on POD 1 in cFNB vs. no-PNB (7 (11%) patients vs. vs. 28 (42.4%), p < 0.001) and POD 2 (2 (3.1%) patients vs. 9 (13.6%), p < 0.046.  No dif. between sFNB and no-FNB. |  | - Earlier ROM 90 degrees in both cFNB and sFNB vs. no-PNB (mean (SD) 2.4 (1.4) days vs. 2.3 (1.4) vs. 3.0 (1.6), p < 0.05)  - No dif. in independent straight leg raise, walking using walking or quad stick, and obstacle clearance  - No dif. in LOS | 1 DVT in FNB. |
| Chan et al.^8^ | RCT | 200 | TKA | Unknown technique – single shot vs. continuous. | *Three groups:*  65 cFNB + ? vs.  69 sFNB + ? vs.  ?  ** Unclear which method of anaesthesia* |  |  |  |  |  |  | - Faster TUG Test at 12 weeks for both sFNB and cFNB compared to no-FNB (mean (SD) 14.3 (7.4) seconds vs. 13.4 (5.6) vs. 11.4 (3.8), p < 0.05). No dif. at 2 weeks  - No dif. in WOMAC score at 2 weeks and 12 weeks.  - No dif. in 6 minutes walking test at 2 and 12 weeks. |  |  |
| Chaudet^9^ | RCT | 55 | Hip fracture | US and/or NS – continuous | 26 cFNB + GA vs. placebo + GA | Total opioid consumption and adverse event rates | - No dif. in total opioid consumption  - Less nausea (8 (31%) patients vs. 17 (59%), p = 0.03).  - No dif. in other side effects | Catheter was accidently removed in 7 patients | No dif. in VAS at any time point | No dif. in IO sufentanil administration |  |  | No dif. in LOS | None related to block |
| Cooke et al.^10^ | RCT | 42 | Fixation tibia plateau fracture | ? - Continuous  ** Unclear which technique* | 21 cFNB + GA vs. GA | Effect on VAS score | No dif. in VAS score at 4, 8, 12, 24, 36, 48, and 72 hr. |  |  | No dif. in total opioid consumption | No if in side-effects |  |  |  |
| Dold et al.^11^ | Retrospective | 96 | Hip arthroscopy | US – single shot | 56 sFNB + GA vs. GA. |  |  |  | - Lower pain score at 60 min in PACU (mean 2.5 vs. 3.7, p = 0.02). No dif. in pain score at 0, 15, 30 and 45 min in PACU.  - Lower maximal pain score at ward (mean 4.0 vs. 5.0, p = 0.18)  ** No dispersion measures shown * Unclear which pain score was used* | - Less IO opioids consumed (mean (SD) 2.7 (4.4) MME vs. 8.1 (5.4), p < 0.0001)  - Less opioid consumption at PACU (mean 2.0 mg MME vs. 4.0, p = 0.025).  - No dif. in oxycodone consumption at ward  ** Unclear whether IV or oral MME* | No dif. In PONV |  | - No dif. in LOS PACU  - 2 patients GA were admitted inpatient for pain management because of inadequate pain control vs. none in FNB group | None related to block |
| Faunø et al.^12^ | RCT | 45 | ACL reconstruction | US – single shot | 23 sFNB + GA or placebo + GA. | VAS score | - Lower VAS score at 1 hr (mean (SD) 2.3 (2.5) vs. 4.3 (3.1), p = 0.02), 2 hr (1.7 (2) vs. 3.7 (2.6), p = 0.01), 3 hr (1.2 (1.1) vs. 2.7 (2.2), p = 0.02), 4 hr (1.2 (1.3) vs. 2.7 (2), p = 0.02), 5 hr (1.3 (1.5) vs. 2.5 (1.5), p = 0.04) and 6 hr (1.3 (1.6) vs. 2.8 (2), p = 0.02). No dif. POD 1 until 6. |  |  | - Less use of fentanyl during first 6 hr after surgery (mean 35 (7) μg vs. 58 (4,) p = 0.02).  - Less use of morphine during first 6 hr (6 (1) mg vs. 10 (2), p = 0.04).  - No dif. in NSAID and acetaminophen during first 14 days. |  |  |  |  |
| Gabriel et al.^13^ | Retrospective | 219.327 | TKA | ? FNB - ?  * *Unclear which technique was used* | 59,833 cFNB or sFNB + GA or spinal vs. GA or spinal |  |  |  |  |  | Less nausea/vomiting (2.0% vs. 4.7% , p < 0.001) |  | Decreased rates of extended PACU stay (62 (1.7%) vs. 1501 (4.7%) patients, p < 0.001) |  |
| Guirro et al.^14^ | RCT | 53 | ACL reconstruction | NS – single shot | 27 FNB + spinal vs. spinal. | VRS | No dif. at 6 hr, 12 hr and 24 hr | 100% |  | No dif. in rescue analgesics asked |  |  |  | Two falls, both FNB group |
| Hadzic et al.^15^ | RCT | *Part 2:*  184 | TKA | US – single shot | 92 sFNB + GA or spinal or epidural vs. placebo + GA or spinal or epidural | Area under the curve for NRS at rest through 72 hr. | Least-squares mean lower (mean (SD) 419 (17) vs. 516 (17), p < 0.001) |  | More patients pain free at 12 hr (p = 0.0038) | - Less total opioids after surgery (76 mg IV MME vs. 103, p = 0.0016).  - Median time to first opioid similar  ** No dispersion measures shown.* | No dif. |  | - Lower OBAS (indicating superior analgesia) at 24 hr (mean 5.6 vs. 7.3, p = 0.0011) and 72 hr (3.1 vs. 4.1, p = 0.007)  - No dif. in proportion patients who were ‘satisfied’ or ‘extremely satisfied’ with their pain control at 72 hr and 30 days.  ** No dispersion measures shown.* | Three falls, all FNB group. Investigators judged that the study drug was related to the fall in one patient |
| Hajian et al.^16^ | RCT | 54 | ACL reconstruction | US – single shot | 27 sFNB + GA vs. GA |  |  |  | - Higher VAS at 20 hr (mean 3.0 vs. 2.3, p = 0.02) and 24 hr (3.2 vs. 2.3, p = 0.02). No dif. at 0 hr, 4 hr, 8 hr, 12 hr, and 16 hr.  ** No dispersion measures shown.* |  | No dif. in side-effects |  | No dif. in patient satisfaction at 12 and 24 hr |  |
| Helsø et al.^17^ | Retrospective | 456 | Hip fracture | NS – continuous | 366 cFNB + GA vs. GA. |  |  |  |  | No dif. in total morphine consumption during admission | No dif. |  | - No dif. in LOS.  - No dif. in mobilization data | No dif. in inhospital falls |
| Kratz et al.^18^ | RCT | 52 | THA | NS – single shot | 26 sFNB + GA vs. GA. | Hemodynamic stability and pain | N.a. |  | -Lower VAS at 6 hr (mean (SD) 2.2 (2.3) vs. 4.9 (1.8), p < 0.0001) and 24 hr (1.7 (2.2) vs. 4.0 (1.9), p < 0.003). No dif. at 30 min, 2 hr. | - Lower cumulative dose of ibuprofen (mean (SD) 69 (200) mg vs. 292 (490), p < 0.05).  - Less piritramide in PACU (mean (SD) 3.3 (2.8) mg vs. 7.3 (4.9), p = 0.0011). |  |  | Shorter LOS PACU (mean (SD) 116 (61) min vs. 157 (47), p = 0.014). |  |
| Krych et al.^19^ | Retrospective | 196 | ACL reconstruction | NS – continuous | 96 cFNB + GA vs. GA. |  |  |  |  |  |  | - No dif. in full return to sport.  - Worse fast extension isokinetic strength at 6 months (78% vs. 85%, p < 0.01). No dif at slow extension, fast flexion and slow flexion isokinetic strenght |  | None related to block. |
| Lomarat et al.^20^ | RCT | 48 | Radiofrequency ablation | US – single shot | 24 FNB + TA vs. TA | Intravenous fentanyl | Lower total dose (mean (SD) 42.7 (26) vs. 107.3 (41) mcg, p < 0.001) |  | - Less IO pain (NRS < 4: 92% vs 33%, NRS 4-7: 8% vs. 54%, NRS > 7, 0% vs. 13%, p < 0001).  - No dif. in pain at 2,6 or 12 hr after surgery |  |  |  | - Quadriceps strength lower 2hr after surgery (weak 79% vs. 0%, p < 0.001)  - No dif. in quadriceps strength 6 and 12 hr after surgery | None related to block. |
| Lovald et al.^21^ | Retrospective | 39,067 | TKA | ? - single shot or continuous  * *Technique unclear* | *Three groups: 3425* cFNB + GA vs. 5370 *s*FNB + GA vs. GA. |  |  |  |  |  |  | - FNB lower risk of readmission at 30, 90 and 365 days (p < 0.001).  - Injection group has higher revision in 30 days (p = 0.015).  - Higher risk for DVT at 30, 90 and 365 days (p < 0.012).  - More knee stiffness at 30, 90 and 365 days (p < 0.014) |  | No significant difference in falls, measured at 30, 90, and 365 days |
| Magnussen et al.^22^ | RCT | 30 | ACL reconstruction | US – single shot | 13 sFNB + *?* vs. *?*  ** Unclear which method of anaesthesia* |  |  |  |  |  |  | - Lower QF-LSI (worse) at 6 weeks (mean dif. -13.4, 95% CI -22.7 to -4.1%, p = 0.005).  No dif. at 6 months  - Lower KOOS score (worse) at 6 weeks (mean dif. -10.4, 95% CI -20.0 to -0.9, p = 0.032). No dif. at 6 months. |  |  |
| Ogawa et al.^23^ | Retrospective + PSM | 103  *PSM: 78* | Hip fracture in patients ≥ 75 years. | US – single shot | 46 sFNB + spinal vs. spinal. | 3 day CAS | *PSM:* Higher (= improved, mean (SD) 8.7 (3.4) vs. 7.3 (2.6), p = 0.048) |  |  |  |  | *PSM:* Higher on POD 2 (mean (SD) 3.1 (1.4) vs. 2.6 (0.9), p = 0.049). | No dif. in LOS. |  |
| Peng et al.^24^ | RCT | 280 | TKA | US + NS – continuous | 140 cFNB + GA vs. GA | Incidence of moderate-to-severe pain 3 months after surgery | - Lower at rest (52 (37.1%) patients vs. 71 (50.7%), p = 0.022)  - No dif. in motion |  | - Lower VAS in motion on POD 7 (median [IQR] 3 [3-4] vs. 4 [4-4], p < 0.0001). No dif. in 24 hr and 48 hr.  - Lower VAS in rest on POD 7 (median [IQR] 3 [2-3] vs. 3 [3-3]. p = 0.031). No dif. in 24 hr and 48 hr. | - Less frequency of PCA-bolus (mean (SD) 2.3 (0.8) vs. 2.6 (0.7), p = 0.003).  - Less frequency of rescue during PCA period (mean (SD) 0.6 (0.8) vs. 1.1 (1.3), p = 0.002). | - Lower frequency of incomplete analgesia in motion (9 (7.1%) patients vs. 21 (17.1%), p = 0.015) | - Lower incidence of chronic pain at rest 6 months (36 (33.0%) patients vs. 54 (52.4%), p = 0.004).  - Lower VAS in rest at 3 months (median [IQR] 2 [1-3] vs. 1 [1-2]), and 6 months 2 [1-3] vs. 1 [1-2], both p < 0.001.No dif. at 12 months.  - No dif. VAS in motion at 3, 6 and 12 months.  - Lower WOMAC scores (= improved) at 3 and 6 months (p = 0.014 and 0.011).  * No dif. at 12 months  ** No scores displayed* | Higher ROM at discharge, 3 months, 6 months, no dif. 12 months. | None related to block |
| Polischuk et al.^25^ | Retrospective | 991 | Hip fracture | Unkown – single shot by emergency staff  * *Technique unclear* | 665 sFNB + ? vs. ?  ** Unclear which method of anaesthesia* | 1 year mortality | No dif. |  |  |  |  | Less likely non-ambulant at 6 months (58 (9%) patients vs. 43 (13%], p = 0.03) and at 12 months (59 (9%) patients vs. 48 (15%), p = 0.005). |  |  |
| Ren et al.^26^ | RCT | 41 | Opening-wedge high tibial osteotomy | US – single shot | 20 sFNB + epidural vs. epidural |  |  | 100% | Lower VAS at 12 hr during rest (mean (SD) 3.5 (1.0) vs. 4.7 (1.1), p < 0.001) and movement (4.5 (0.6) vs. 5 (0.8), p = 0.04). No dif. at 6 and 18 hr and POD 1, 3, 7, 14 | No dif. in consumption of opioids or NSAIDs | No dif. in nausea, vomiting and dizziness. |  | No dif. in quadriceps strength at 24 and 48 hr after surgery |  |
| Rowlands et al.^27^ | RCT | 111 | Femur fracture ≥ 70 years | US – continuous | 55 cFNB + spinal or GA vs. spinal or GA | CAS and cumulative dynamic pain score from POD 1 to 3 | - No dif. in CAS  - No dif. In cumulative dynamic pain scores. | 2 patients catheter resites, 4 catheters dislodged | Lower cumulative pain score at rest (median [IQR] 2 [0-5] vs. 5 [0.5 – 6.5], p = 0.043). |  | No dif. in nausea and vomiting. |  | - No dif. in LOS.  - No dif. in EQ-5D score at 3 or 30 days | None related to block |
| Sahin et al.^28^ | RCT | 104 | TKA | US – single shot | 51 sFNB + spinal vs. placebo + spinal | VRS 48 hr | Lower at rest and movement at 4 hr, 8 hr, 12 hr, 24 hr, and 48 hr (all p < 0.001). No dif. at 1 hr.  **No values or dispersion measures shown* |  |  | Less IV morphine use at 24 hr (mean (SD) 12.4 (5.3) mg vs. 25.6 (7.2), p < 0.001) and 48 hrs (18.7 (9.6) mg vs. 39.6 (11.2), p < 0.001) | No dif. in nausea, vomiting, pruritus, dizziness |  | Lower patient satisfaction score (= improved) (median [range] 2 [1-4] vs. 4 [1-4], p < 0.01). | None related to block |
| Sengoku et al.^29^ | Retrospective | 107 | ACL reconstruction | US – single shot | 66 sFNB + GA vs. GA. |  |  |  |  |  |  | No dif. in knee extensor en flexor strength at 3 or 6 months |  |  |
| Singh et al.^30^ | RCT | 60 | Femur fracture | NS – single shot | 30 sFNB + spinalepidural vs. spinalepidural |  |  |  | Lower VAS (p <0.001)  * *Not clear at which time-point* | - Less epidural top-ups after surgery (mean 2.8 vs. 3.7, p < 0.001).  ** No dispersion values shown* | No dif. in nausea and vomiting |  |  | None related to block |
| Thomas et al.^31^ | Retrospective + matching | 54 | TKA | ? – continuous  * *Unknown technique* | 27 cFNB + ? vs. ?  ** Unclear which method of anaesthesia* |  |  |  | Lower VAS over first 3 postoperative days (mean (SD) 2.7 (1.2) vs. 3.5 (1.1), p = 0.01). | No dif. in narcotic use |  |  | No dif. in achievement of physical therapy mobilization goal |  |
| Unneby et al.^32^ | RCT | 266 | Hip fracture in patients ≥ 70 years | NS – single shot | 129 sFNB + ? vs. ?  ** Unclear which method of anaesthesia* |  |  |  | Lower VAS at 2 hr (median [IQR] 2 [0-3.5] vs. 3 [2-5], p = 0.003). No dif. at 6, 12, and 18 hr | - Lower IV opioid consumption at ward (mean (SD) 2.3 (4) mg. vs. 5.7 (5.2), p < 0.001).  - Lower oral opioid consumption at ward (mean (SD) 2.1 (4.1) mg vs. 3.6 (6.4), p = 0.017). |  |  |  | None related to block |
| Unneby et al.^33^ | RCT | 236 | Hip fracture in patients ≥ 70 years | NS – single shot | 116 sFNB + ? vs. ?  ** Unclear which method of anaesthesia* | Postoperative delirium | No dif. |  | No dif. in VAS on POD 3-5 | No dif. consumed opioids POD 1-5 | More drugs adverse effects (8 patients (7.1%) vs. 0 (0%), p = 0.003). |  | - No dif. in LOS  - No dif. in MMSE-score, OBS, GDS-15 between POD 3-5 | None related to block |
| Uysal et al. ^34^ | RCT | 91 | Femur fracture | US + NS – intermittent | 46 iFNB + spinal-epidural combined vs. spinal-epidural combined |  |  |  | No dif. in VAS at 1, 4, 12 and 24 hr |  |  |  |  |  |
| Wiesmann et al.^35^ | RCT | 80 | THA | NS - single shot | 40 sFNB + GA vs. GA |  |  |  | Lower VAS at 6 hr (mean (SD) 2.6 (2.3) vs. 4.5 (2.3), p < 0.001) and 24 hr (2.1 (2.3) vs. 3.9 (2.3), p < 0.001). No dif. at 0, 0.5, and 2 hr | - Less PACU piritramide consumption (mean (SD) 3.5 (2.9) IV mg vs. 7.7 (4.9), p < 0.001)  - Less ibuprofen consumed ward (mean (SD) 65.0 (183.4) mg vs. 320 (481.0), p = 0.0024) | No dif. in PONV |  | Earlier discharge from PACU (mean (SD) 116 (40) min vs. 152 (47), p < 0.001) |  |
| Wu et al.^36^ | RCT | 60 | TKA | US and NS – continuous | 30 cFNB + spinal vs. spinal |  |  |  | No dif. in VAS at rest or mobilization on POD 0 to 3 | - Less opioid consumption on POD 0 (mean (SD) 3.1 (2.1) IV MME vs. 15.5 (8.9), p < 0.0001) and POD 1 (mean (SD) 6.1 (0.9) IV MME vs. 10.7 (10.1), p = 0.021).  - More opioid consumption on POD 3 (mean (SD) 3.4 (1.6) IV MME vs. 1.6 (2.9), p = 0.006). No dif. on POD 2 | - Less nausea and/or vomiting (8 (27%) patients vs. 19 (63%), p < 0.05).  - Less dizziness (5 (17%) patients vs. 12 (40%), p < 0.05). | No dif. in Knee Society score and Functional score at 6 weeks, 3 and 6 months | - Longer in PACU (mean 62 min vs. 46, p = 0.014).*  - Improved satisfaction (mean (SD) 8.3 (2.0) vs. 7.2 (2.3), p = 0.05).  - More mobilization on POD 1 (25 patients (83%) vs. 15 (50%), p = 0.012).  - Earlier independent walking (POD 6 vs. 8, p = 0.012)  - No dif. in LOS.  ** No dispersion values shown* | 5 patients DVT (2 in block group). |
| Xing et al.^37^ | RCT | 50 | Hip arthroscopy | US – single shot | 27 sFNB + GA vs. placebo + GA. | Cumulative opioid consumption first 24 hr | No dif. | 100% | Lower VAS at 0.5 hr (mean (SD) 5.6 (2.4) vs. 7.1 (1.9), p = 0.009), 1 hr (4 (1.9) vs. 5.7 (2.3), p = 0.004), 2 hr (3.2 (1.7) vs. 4.8 (2.1), p = 0.003), 4 hr (3 (1.5) vs. 4.3 (1.9), p = 0.006) and 6 hr (2.3 (1.3) vs. 4.1 (1.9), p < 0.001). No dif. at 1.5, 24, 48 hr, and 7 days | Less postoperative opioid consumption at 48 hr (mean (SD) 10.9 (12.5) oral MME vs. 26.6 (24.6), p = 0.006). No dif. at 24 hr, and POD 7 | - No dif. in PONV  - No dif. in constipation, itching, pain, and bruising |  | - No dif. in PACU time  - No dif. in satisfaction | Higher risk of falls in first 24 hr (6 patients (22.5%) vs 0 (0%), p = 0.025) due to muscle weakness. |
| Yan et al.^38^ | Retrospective | 103 | TKA | US – continuous | 52 cFNB + GA vs. GA |  |  |  | - Lower VAS score while resting a 4 hr, 6 hr, 12 hr, 24 hr and 48 hr after treatment (all p < 0.05).  - Lower VAS score active and passive at 4 hr, 6 hr, 12 hr, 24 hr and 48 hr after treatment (all p < 0.05).  **No values mentioned.* |  | - Less PONV on POD 1 (2 patients (3.8%) vs. 16 (31.4%), p < 0.001 and POD 4 (1 patient (1.9%) vs. 8 (15.7%), p = 0.029.  - No dif. in PONV on POD 7.  - Less other side effects (3 patients (5.8%) vs. 15 (29.4%), p < 0.05. |  | - Greater active motion knee joint at 4, 6, 12, 24 and 48 hr, all p < 0.05*  - Higher muscle strength scores at 4, 6, 12, 24 and 48 hr, all p < 0.05*  - Lower incidence of POCD on POD 1 and 4, p < 0.05. No dif. POD 7.  - Higher MMSE scores on POD 1, 4, 7, p < 0.05  - Higher patient satisfaction (mean (SD) 95.7 (2.9) vs. 75.1 (2.3), p < 0.05).  * *No values mentioned* |  |
| Yao et al.^39^ | Retrospective | 694 | TKA | US – single shot | 250 FNB + GA vs. GA | Incidence of chronic pain | No dif. at 3, 6 and 12 months |  | Lower VAS on POD 1 at rest (mean (SD) 2.3 (0.9) vs. 2.7 (1), p < 0.001) and motion (3.2 (1) vs. 4.1 (1.0), p < 0.001) and POD 2 at rest (1.6 (0.8) vs. 1.9 (0.8), p < 0.001) and motion (3.1 (1) vs. 3.6 (0.8), p < 0.001). | Less use of rescue anaesthesia on POD1 (32 (12.0%) patients vs. 114 (24.3%), p = 0.001). No dif. on POD 2 |  | No dif. in VAS during rest and motion at 3, 6, and 12 months |  |  |
| Zhang et al.^40^ | RCT | 60 | TKA in patients ≥ 75 years | NS - single shot. | *Three groups*  20 sFNB + LMA + GA vs. 20 sFNB + tracheal intubation + GA vs. GA. |  |  |  | - Lower VAS at rest at 6h both FNB groups vs. no-FNB (median [IQR] 2 [1.8-3] vs. 2 [1.8-3] vs. 3 [3-4], p < 0.05), at 24 hr (2 [1-3] vs. 2.5 [2-3] vs. 4 [3-4.3], p < 0.05).  - Lower VAS during training at 24h (median [IQR] 3 [2-3.3] vs. 3 [2-4] vs. 4.5 [4-5.3], p < 0.05), and 48 hr (2.5 [2-3] vs. 3 [2 – 3] vs. 4 [3-4.25], p < 0.05) | - Less total dose of PCA pump (mean (SD) 75.8 (2.0) ml vs. 75.6 (1.3) vs. 79.2 (1.6), p < 0.01)  - Less use of parecoxib (mean (SD) 32 (12.1) mg vs. 30 (12.4) vs. 136, p < 0.01).  - Less use of IO remifentanil (mean (SD) 1 (0.3) mg vs. 1 (0.3) vs. 1.3 (0.3), p < 0.01).  - Less use of IO fentanyl (mean (SD) 0.2 (0.1) mg vs. 0.2 (0.1) vs. 0.4 (0.1), p < 0.01).  - Less use of IO propofol (mean (SD) 274 (31) mg vs. 287 (29) vs. 395 (41), p < 0.01). | No dif. |  |  |  |

**Abbreviations:** N = number of patients, PONV = postoperative nausea and vomiting, US = ultrasound, sFNB = single shot femoral nerve block, TA = tumescent anaesthesia, dif. = difference, VAS = visual analogue scale, RCT = Randomised controlled trial, TKA = total knee arthroplasty, NS = nerve stimulation, GA = general anaesthesia, cFNB = continuous femoral nerve block, ROM = Range of motion, LOS = length of stay, TUG = Timed Up & Go Test, WOMAC = Western Ontario and McMaster Universities Arthritis Index, POD = postoperative day, MME = milligram morphine equivalent, IV = intravenous, THA = total hip arthroplasty, ACL = anterior cruciate ligament, FIM = Functional Independence Measure, bolus DVT = deep venous thrombosis, IO = intraoperative, PACU = post anaesthetic care unit, NSAID = non-steroidal anti-inflammatory drugs, VRS = verbal rating scale, NRS = numeric rating scale, OBAS = overall benefit of analgesic score, QF-LSI = quadriceps femoris strength limb symmetry indices, KOOS = Knee injury and osteoarthritis outcome, PSM = propensity score matching, CAS = cumulated ambulation score, MMSE = mini mental state examination, OBS = organic brain syndrome scale, GDS-15 = geriatric depression scale, iFNB = intermittent femoral nerve block, POCD = postoperative cognitive dysfunction, LMA = laryngeal mask airway, PCA = patient-controlled analgesia

**Saphenous nerve block (adductor canal block)**

| **Author** | **Design** | **N** | **Surgery** | **Technique** | **Anaesthetics** | **Primary outcome** | **Result primary outcome** | **Success rate** | **Pain** | **Analgesia** | **PONV or other side effects** | **Long-term outcomes** | **Other (quality of recovery, satisfaction, functional recovery, hospital stay)** | **Complications** |
| --- | --- | --- | --- | --- | --- | --- | --- | --- | --- | --- | --- | --- | --- | --- |
| Agarwala et al.^41^ | Before/after study | 271 | TKA | US – single shot | 151 sACB + LIA + spinal vs. LIA + spinal | VAS at 24 hour at rest and on movement | - Lower at rest (mean (SD) 1.2 (1) vs. 1.7 (0.9), p < 0.001)  - Lower at movement (mean (SD) 2.1 (0.9) vs. 2.4 (1.1), p = 0.01) |  | - Lower at rest at 0 hr (mean (SD) 2 1.7 vs. 2.8 (1.3), p < 0.001), 6 hr (1.6 (1.2) vs. 2.1 (1), p = 0.008), 12 hr (1.4 (1) vs. 1.7 (0.9), p = 0.017)  - Lower at movement at 0 hr (mean (SD) 2.2 (1.9) vs. 3.1 (1.5), p < 0.001), 6 hr (2 (1.3) vs. 2.6 (1.1), p = 0.001), 12 hr (1.9 (1) vs. 2.2 (1), p = 0.01) |  |  |  | No dif. in sitting, standing, and walking with walker within 24 hr | None related to block |
| Andersen et al.^42^ | RCT | 40 | TKA | US – continuous | 20 cSB + LIA + spinal vs. placebo + LIA + spinal | Worst pain during movement on POD 0 | Lower (median [IQR] 3.0 [1.3-6] vs. 5.5 [3-8], p < 0.05). |  | - No dif. in worst pain during movement on POD 1 and 2  - Lower pain at rest on POD 0 (median [IQR] 2.0 [0-3] vs. 4.0 [1.3-6.8, p = 0.032). No dif. on POD 1 and 2.  - Longer time till VAS > 3 (median [IQR] 10.5 [0.5-48] hours vs. 3.4 [0.5-24], p = 0.011) | No dif. in cumulated morphine consumption | No dif. |  | - More patients able to ambulate on POD 0 (n = 20 vs. 13, p = 0.004)  - Less sleep disturbance (5 patients on POD 0 (25% vs. 11 55%, p = 0.038). No dif. POD 1 and 2.  - No dif. in LOS | 1 patient developed hematoma and paraesthesia after catheter removal cSB, the patient was treated with gabapentin for 4 months until the paraesthesia subsided |
| Arumugam et al.^43^ | RCT | 70 | Arthroscopic knee | US – single shot | 35 sACB + spinal vs. spinal | 24 hr IV morphine consumption | Less (mean (SD) 8.6 (1) vs. 21.9 (5) mg, p < 0.001) |  | - Longer duration of analgesia (mean (SD) 366 (54) vs. 150 (23), p < 0.001)  - Lower VAS at rest and in flexion between 3 and 24 hr, similar first 2 hr*  ** No values mentioned* |  | No dif. in adverse effects |  |  | None related to block |
| Brush et al.^44^ | RCT | 80 | Arthroscopic meniscus repair or partial meniscectomy | Landmark (surgeon) – single shot | 40 sSB + general or sedation vs. no SB + ?  * *Unclear which anesthetic method* | VAS immediate postoperative | No dif. |  | No dif. in VAS POD 1 and 7 | No dif. in opioids in MME after surgery |  |  |  |  |
| Espelund et al.^45^ | RCT | 71 | Arthroscopic knee | US – single shot | 36 sACB + GA vs. placebo + GA | VAS during standing at 2 hr. | No dif. |  | - No dif. in VAS at rest or during standing 0-24 hour - No dif. in VAS after 5 meter walk 2-24 hour | Less ketobemidone consumption 0-2 hr (median [IQR] 0 [0-2.5] IV mg vs. 2.5 [0-5], p = 0.01). No dif. 2-24 hr. | No dif. in opioid side effects |  |  | None related to block |
| Espelund et al.^46^ | RCT | 49 | ACL reconstruction | US – single shot | 25 sACB + GA vs. placebo + GA | VAS during standing at 2 hr. | No dif. | 100% | - No dif. in VAS at rest and standing 0-24 hour  - No dif. in VAS after 5meter walk 2-24 hour | No dif. in ketobemidone consumption 0 – 24 hour | No dif. in opioid side effects |  |  | None related to block |
| Gwam et al.^47^ | Retrospective | 110 | TKA | US – single shot | 65 sACB + MPA + ? vs. MPA + ?  ** Unclear which method of anaesthesia* |  |  |  | No dif. in VAS score | No dif. in opioid consumption at POD 0, 1, 2, 3 |  |  | - No dif. in discharge status (i.e. home vs. rehabilitation).  - No dif. in LOS |  |
| Goytizolo et al.^48^ | RCT | 111 | TKA | US – single shot | 55 sACB + PAI + spinal vs. PAI + spinal | Time until meeting discharge criteria | No dif. |  | - No dif. in NRS at spinal resolution, at 24 hr and 48 hr (at rest, ambulation, flexion, and physical therapy).  - Lower worst pain in last 24 hr (mean 5.3 (2.9) vs. 6.7 (2.5), p = 0.041). | No dif. in opioid use 0 -48 hr | No dif. in opioid side-effects |  | No dif. in patient satisfaction |  |
| Gudmundsdottir^49^ | RCT | 69 | TKA | US – continuous | 35 cACB + LIA + spinal vs. placebo + LIA + spinal | Peak NRS during morning physio session on POD 1. | No dif. |  | Lower NRS at rest on morning POD 1 (median [range] 1 [0-6] vs. 2 [0-5], p = 0.04). No dif. on POD 2. | - No dif. in total morphine consumption  - No dif. in time to first additional pain medication |  |  | No dif. in ambulation-ability test on POD 1 & 2. |  |
| Hanson et al.^50^ | RCT | 48 | Arthroscopic medial meniscectomy | US – single shot | 24 sACB + GA vs. sham + GA | Resting NRS pain upon arrival to the PACU. | Lower (mean dif. 1.7, 95% CI 0.7 to 2.7 vs. 3.3, 95% CI 2.3 to 4.2, p = 0.03). |  | Lower NRS at PACU discharge, 12, 18 and 24 hr (p < 0.05).  ** No NRS values mentioned* | - Less fentanyl used (mean (SD) 62 (35) μg vs. 40 (47), p = 0.011).  - Less total opioid consumption over 24 hr (mean dif. 45, 95% CI 29.5 to 60.2 oral MME vs. 72, 95% CI 56.5 to 87.2, p = 0.016). |  |  |  | None related to the block |
| Jæger et al.^51^ | RCT | 30 | Revision TKA | US – single shot | 14 sACB + GA vs. placebo + GA. | VAS during knee flexion at 4 hr. | Lower (mean (SD) 52 (22) vs. 71 (25) mm, p = 0.04). |  | No dif. in VAS in rest and during flexion at 1, 2, 6, 8, or 24 hr. | No dif. in cumulative morphine consumption | No dif. |  |  | None related to block |
| Johns et al.^52^ | Retrospective | 458 | TKA | *? –* 50% single shot and 50% continuous  * *Unknown technique* | 138 sACB or cACB + ? vs. ?  ** Unclear which method of anaesthesia* |  |  |  |  | Less opioids on discharge (median [range] 30 [0 – 150] oral MME vs. 45 [15-110], p < 0.001). |  |  | - Longer LOS (mean (SD) 5.0 (2) days vs. 4.2 (1.5), p < 0.0001).  - No dif. in ROM. | . |
| Kejriwal et al.^53^ | RCT | 60 | ACL reconstruction | US – single shot | 30 sSB + GA + intra-articular and local anaesthetic vs. GA + intra-articular and local anaesthetic | Postoperative patient satisfaction | No dif. | 100% | - Lower VAS score at 4 hr (mean 1.9 vs. 3.0, p = 0.037). No dif. other time points (0, 8, 24 hr)  ** No dispersion measures mentioned* | - No dif. total opiate consumption.  - Less oral opiate need (mean 3.7 vs. 1.1 mg p = 0.04).  ** No dispersion measures mentioned* | No dif. in nausea and/or vomiting. |  | No dif. in patient satisfaction |  |
| Laksono et al.^54^ | RCT | 30 | ACL reconstruction | ? – single shot  * *Unknown technique* | 15 sACB + spinal vs. spinal |  |  |  | - Lower NRS at rest at 6 hr (mean (SD) 0.6 (0.3) vs. 1.4 (0.6)), 12 hr (0.5 (0) vs. 1.6 (0.6)), 18 hr (0.6 (0.3) vs. 1.6 (0.7)), POD 1 (0.6 (0.3) vs. 1.6 (1)), and 2 (1 (1.2) vs. 2.1 (0.7)), all p< 0.001. No dif. POD 3.  - Lower NRS at movement at 6 hr (mean (SD) 0.6 (0.3) vs. 1.6 (0.4)), 12 hr (0.5 (0) vs. 1.7 (0.4)), 18 hr (0.6 (0.3) vs. 1.7 (0.8)), POD 1 (0.6 (0.3) vs. 1.5 (0.7)), and 2 (2.2 (0.7) vs. 1.2 (1.6)), all p< 0.001. No dif. POD 3. | No dif. in need for rescue analgesia |  |  | No dif. in LOS |  |
| Lan et al.^55^ | RCT | 42 | Medial unicondylar knee arthroplasty (55-75 year) | US – Continuous | 22 cACB + LIA + spinal vs. placebo + LIA + spinal | NRS with active knee flexion at 24 hr | Lower (median [IQR] 3 [2.8-4.3] vs. 5 [4-6], p < 0.001). |  | - Longer time until NRS > 3 (median [IQR] 18.5 [4-46] hr vs. 10.0 [3-24] hours, p = 0.002).  - Lower NRS at rest and movement at 8, 12, 24, 48 hr  * *No values mentioned* | Less IV morphine use at 24-48 hrs (mean (SD) 15.6 (10.5) IV MME vs. 27.2 (21.5), p = 0.039). No dif. 0-24 hr |  |  | - No dif. in quadriceps muscle strength  - More distance ambulated on POD 1 (mean 37.2 (32.2) vs. 19.7 (22.1) meter, p = 0.046) and POD 2 (59.5 (28.3) meter vs. 33.4 (20.8), p = 0.002).  - No dif. in patient satisfaction at 24 and 48 hr. | None related to block. |
| Leung et al.^56^ | RCT | 70 | TKA | US – Continuous | 38 cACB on POD 1 + (spinal-)epidural vs. sham on POD 1 + (spinal-)epidural | Total opioid consumption | Less at 20 hr (mean (SD) 73.9 (38) MME vs. 96.5 (47), p = 0.03).  ** Unclear whether oral or IV MME* |  | Less pain at 20 hr on VAS scale (mean (SD) 28.6 (1.4) mm vs. 36.4 (18), p = 0.04). No dif. at 12 hr |  |  | Improved WOMAC score at 3 weeks (mean (SD) 37.8 (13) vs. 29.1 (15), p = 0.04). No dif. at 6 weeks | - No dif. in LOS  - Decreased ROM on POD 1 (mean (SD) 40.6 (14) degrees vs. 51.4 (17) degrees, p = 0.006). No dif. POD 2, and 3 or 6 weeks.  - No dif. in ambulation distance POD 1 or 2 | None related to block |
| Nader et al.^57^ | RCT | 40 | TKA | US – single shot | 20 sACB + spinal + LIA vs. placebo + spinal + LIA | Total opioid consumption first 36 hr | Less (median [IQR] 48 [39 - 61] IV MME vs. 60 [49 - 85], p = 0.03). |  | - Lower NRS with first report (median [IQR] 4 [2-5] vs. 6 [5-7], p = 0.04).  - No dif. in time to first pain |  | No dif. in adverse effects | - No dif. in NRS (rest and activity) at 3 weeks  - No dif. in opioid consumption at 3 weeks  - No dif. in ROM at 3 weeks | - Improved patient satisfaction (median [IQR] 8.5 (7-10) vs. 6.5 (5-9.5), p = 0.04).  - No dif. in physical therapy milestones on POD 0 and 1 (i.e. leg raise, get out of bed, walk, climb stairs) |  |
| Nazemyanyazdi et al.^58^ | RCT | 80 | TKA | US – single shot | 40 sSB + spinal vs. spinal |  |  |  | - Higher VAS at 12 hr (mean (SD) 5.3 (0.9) vs. 4 (0.8)) and 24 hr (4.6 (1) vs. 3 (0.8)), both p < 0.05.  - No dif. at 2 and 6 hr. | More diclofenac use within 24 hr (262 vs. 87 mg, p < 0.001) | Less nausea (30% vs. 55%) and vomiting (2.5% vs. 22.5%), both p < 0.001, within 24 hr |  |  |  |
| Nicolino et al. ^59^ | RCT | 70 | TKA | US – Single shot | 34 sSB + epidural + sedation + intra-articular infiltration vs. epidural + sedation + intra-articular infiltration |  |  |  | - More patients pain free at 3-6 hr (33 (91.2%) patients vs. 17 (48.6%), p = 0.001), 6 hr (91.2% vs. 54.3, p = 0.001).  - Less pain 3-6 hr (mean (SD) 1.5 (1.7) vs. 3.8 (2.7), p = 0.01), 6 hr (2.4 (1.9) vs. 3.6 (2.4), p = 0.02), 12 hr (2.4 (1.9) vs. 3.6 (2.4), p = 0.02), and 8 hr after rehabilitation session (3.4 (1.4) vs. 4.5 (1.9), p = 0.01). No dif. at 24, 48 hr and at discharge. | - Less use of rescue morphine in PACU (mean (SD) 0.1 (0.4) rescues vs. 0.7 (0.9), p = 0.002).  - No dif. in total amount of morphine |  |  |  | None related to block. |
| Padki et al.^60^ | Retrospective | 135 | TKA | US – single shot | *Three groups:* 45 sACB by anaesthesist + GA or spinal vs. 45 sACB by surgeon GA or spinal vs. 45 GA or spinal |  |  |  | No dif. in VAS at rest or after ambulation POD 1 |  |  |  | - No dif. in ambulation, flexion, extension, active straight leg raise POD 1  - No dif. in LOS |  |
| Poon et al.^61^ | Retrospective | 652 | TKA | US – Single shot | *Three groups: 222* sACB (pre-op) + GA vs. 149 sACB (post-op) + GA vs. 281 GA |  |  |  | Both ACB lower VAS compared to noACB at discharge PACU (median [IQR} 2 [2 – 2] vs. 1 [1 – 1] vs. 4 [4 – 4], p < 0.001) at the ward in rest (2 [1 – 2] vs. 1 [1 – 2] vs. 3 [3 – 5], p < 0.001) and movement (2 [2 – 3] vs. 2 [1 – 2] vs. 3 [3 – 5], p < 0.001) | - No dif. in opioid consumption in PACU  - Lower opioid consumption both ACB groups at ward (median [IQR] 0.1 [0.1-0.1] MME/kg vs. 0.1 [0.09- 0.1] vs. 0.1 [0.1 - 0.2], p 0.045.  - No dif. in sevoflurane IO  ** Unclear whether IV or oral MME* | No dif. in PONV or dizziness |  | - Shorter LOS (median [IQR]. 4.0 [3.5-5.0] days vs. 4.0 [3.5- 5.0] vs 4.5 [3.5 - 5.0], p = 0.004).  - No dif. in satisfaction | None related to block. |
| Rames et al.^62^ | Retrospective | 693 | TKA | US – single shot | 624 sACB + PAI + ? vs. PAI + ?  ** Unclear which method of anaesthesia* |  |  |  |  | No dif. in total postoperative morphine use |  |  | - Improved ambulation on POD 1 (mean (SD) 75.8 (49.0) ft vs. 59.9 (34.3), p = 0.008).  - Shorter LOS (mean (SD) 34.8 (16.9) hr vs. 40.6 (17.4), p = 0.01). |  |
| Rousseau-Saine et al.^63^ | RCT | 60 | TKA | US – continuous | 30 cACB + spinal vs. sham + spinal | Extensor muscle strength at 6 weeks | No dif. | At POD 1 (100%) and POD 2 (87%). | - Less pain at rest on POD 1 (median [IQR] 3.8 [2.0-4.5] vs. 5.4 [4.0-6.4], p = 0.002). No dif. on POD 2.  - Less pain at maximum extensor muscle strength effort at POD 1 (median [IQR] 6.5 [4.3-8.0] vs. 8.7 [6.7-9.5], p = 0.004) and POD 2 (6.0 [4.6-7.9] vs. 8.0 [7.0-8.3], p = 0.008). | - Less opioid consumption on POD 1 (median [IQR] 22 [11-29] IV MME vs. 34 [23-56], p = 0.012) and POD 2 (15 [8-33] IV MME vs. 30 [20-41], p = 0.032). |  | No dif. in KOOS score at 6 weeks | - Improved extensor muscle strength POD 1 (median [IQR] 15 nm [8-26] vs. 9 nm [4-17], p = 0.042). No dif. on POD 2.  - No dif. in LOS  - No dif. in patient satisfaction | 1 patients had a mild incomplete motor block in a distribution similar to a femoral block, recovered shortly after cACB was removed |
| Sim et al.^64^ | RCT | 35 | Medial open wedge high tibial osteotomy | US – Single shot | 19 sACB + GA vs. placebo + GA | VAS | Lower VAS in the first 12 hr (p = 0.04).* No dif. after 12 hr.  ** No values mentioned.* |  |  | - Less opioid consumption in first 12 hr (16.7% vs. 70%, p = 0.017).  - Less opioid injections within 72 hr (8.3% vs. 50%, p = 0.043). |  | No dif. in quadriceps strength, ROM, time to straight leg raising at 2 weeks, and 3 months. | No dif. in quadriceps strength, ROM, time to straight leg raising at 72 hr | None related to block |

**Abbreviations:** N = number of patients, PONV = postoperative nausea and vomiting, TKA = total knee arthroplasty, US = ultrasound, sACB = single shot adductor canal block, LIA = local infiltration analgesia, VAS = visual analogue scale, dif. = difference, cSB = continuous saphenous nerve block, sSB = single shot saphenous nerve block, POD = postoperative day, IV = intravenous, MME = morphine equivalent dose, GA = general anaesthesia, ACL = anterior cruciate ligament, MPA = multimodal periarticular analgesia, LOS = length of stay, PAI = periarticular injection, NRS = numeric rating scale, cACB = continuous adductor canal block, ROM = Range of motion, WOMAC = Western Ontario and McMaster Universities Arthritis Index, PACU = post anaesthetic care unit, IO = intraoperative, KOOS = Knee injury and osteoarthritis outcome, KES = knee extensor muscle strength

**Sciatic nerve block**

| **Author** | **Design** | **N** | **Surgery** | **Technique** | **Anaesthetics** | **Primary outcome** | **Result primary outcome** | **Success rate** | **Pain** | **Analgesia** | **PONV or other side effects** | **Long-term outcomes** | **Other (quality of recovery, satisfaction, functional recovery, hospital stay)** | **Complications** |
| --- | --- | --- | --- | --- | --- | --- | --- | --- | --- | --- | --- | --- | --- | --- |
| Danisan et al.^65^ | RCT | 60 | Endovascular treatment for below the knee occlusions | US – single shot | 30 sSCNB vs. Fentanyl |  |  | 100% | - Lower IO VAS score (median [range] 0 [0-30] vs. 70 [20-100], p < 0.001)  - Lower IO FLACC scale (median [range] 0 [0-2] vs. 6 [3-10], p < 0.001). | Lower fentanyl consumption (median [range] 0 [0, 0] IV μg vs. 100 [50 – 200]). | Apnea in 3 patients (fentanyl group) |  |  | None related to block |
| Li et al.^66^ | RCT | 120 | Calcaneal fracture | US – single shot | 60 sSCNB + spinal vs. Spinal | VAS 8 hr after surgery | Lower (mean (SD) 0 vs. 5.3 (0.7), p < 0.001). |  | - Lower VAS at 4 hr (mean (SD) 0 vs. 2.1 (1.7)), 8 hr (0 vs 5.3 (0.7)), 12 hr (1.1 (0.8) vs. 5.3 (0.9)), and 16 hr (1.9 (1.2) vs. 3.6 (0.6), all p < 0.001).  - No dif. at 0, 24 and 48 hr. | - Longer time until first press PCA (mean (SD) 15.6 (3.0) hr vs. 4.3 (0.6), p < 0.05)  - Longer use PCA (mean 49.6 (0.2) hr vs. 47.9 (0.3), p = 0.019).  - Less PCA pressing (1.8 (0.9) times vs. 8.5 (1.1), p < 0.05).  - Less rescue analgesia (0.03 (0.2) vs. 0.2 (0.4), p < 0.05) | - Less nausea (2 (3%) patients vs. 8 (11%), p = 0.047).  - No dif. In vomiting and pruritus |  | - Improved patient satisfaction (98.3% vs 81.7%, p = 0.002).  - Improved surgeon and nurse satisfaction (96.7% vs. 85%, p = 0.027). |  |
| Makkar et al.^67^ | RCT | 30 | Traumatic lower limb amputation | US – single shot | 15 sSCNB + GA vs. placebo + GA | Incidence of chronic pain at 6 months | No dif. |  |  | - Less total morphine consumption (mean (SD) 13.4 (3.4) vs. 5 (4) IV mg, p < 0.001).  - Less PCA-presses (median [IQR] 3 [2-6 vs. 14 [11-15, p < 0.001) | Lower PONV score at arrival PACU (median [IQR] 0 [0-0] vs. 2 [0-2]), and at 6 hour after surgery (0 [0-1] vs. 1 [0-2]), | No dif. in intensity of phantom pain and stump pain at 1 month |  |  |
| Schmitt et al.^68^ | Prospective | 27 | Foot surgery in patients with Charcot-Marie-Tooth disease | NS – intermittent | 17 iSCNB + GA or spinal vs. GA or spinal |  |  |  |  | No dif. |  |  |  | None related to block. |

**Abbreviations:** N = number of patients, PONV = postoperative nausea and vomiting, RCT = randomised controlled trial, US = ultrasound, sSCNB = single shot sciatic nerve block, IO = intraoperative, VAS = visual analogue scale, FLACC = face, legs, activity, cry, consolability, IV = intravenous, PCA = patient-controlled analgesia, GA = general anaesthesia, NS = nerve stimulator

**Psoas compartment block**

| **Author** | **Design** | **N** | **Surgery** | **Technique** | **Anaesthetics** | **Primary outcome** | **Result primary outcome** | **Success rate** | **Pain** | **Analgesia** | **PONV or other side effects** | **Long-term outcomes** | **Other (quality of recovery, satisfaction, functional recovery, hospital stay)** | **Complications** |
| --- | --- | --- | --- | --- | --- | --- | --- | --- | --- | --- | --- | --- | --- | --- |
| Green et al.^69^ | RCT | 53 | THA | Landmark (surgeon delivered) – single shot | 26 PCB + spinal vs. spinal |  |  |  | Lower pain score at 2 hr (mean 0.2 vs. 1.2), 10 hr (1.3 vs. 3.4), 20 hr (0.1 vs. 1.2), all p < 0.01) * *No dispersion values shown*  ** *Unclear which scoring system was used* | - No dif. in type of analgesia sought  - More time to first analgesia (324 vs. 260 min, p = 0.005) * *No dispersion values shown ** Unclear if median or mean* |  |  | No dif. in mobilisation. |  |

**Abbreviations:** N = number of patients, PONV = postoperative nausea and vomiting, RCT = randomised controlled trial, THA = total hip arthroplasty, PCB = psoas compartment block, dif. = difference

**References**

1. Al Wahbi AM. Evaluation of pain during endovenous laser ablation of the great saphenous vein with ultrasound-guided femoral nerve block. Vasc Health Risk Manag. 2017;13:305-9.

2. Angers M, Belzile É L, Vachon J, Beauchamp-Chalifour P, Pelet S. Negative Influence of femoral nerve block on quadriceps strength recovery following total knee replacement: A prospective randomized trial. Orthop Traumatol Surg Res. 2019;105(4):633-7.

3. Arsoy D, Gardner MJ, Amanatullah DF, Huddleston JI, 3rd, Goodman SB, Maloney WJ, et al. Continuous Femoral Nerve Catheters Decrease Opioid-Related Side Effects and Increase Home Disposition Rates Among Geriatric Hip Fracture Patients. J Orthop Trauma. 2017;31(6):e186-e9.

4. Arsoy D, Huddleston JI, 3rd, Amanatullah DF, Giori NJ, Maloney WJ, Goodman SB. Femoral Nerve Catheters Improve Home Disposition and Pain in Hip Fracture Patients Treated With Total Hip Arthroplasty. J Arthroplasty. 2017;32(11):3434-7.

5. Astur DC, Aleluia V, Veronese C, Astur N, Oliveira SG, Arliani GG, et al. A prospective double blinded randomized study of anterior cruciate ligament reconstruction with hamstrings tendon and spinal anesthesia with or without femoral nerve block. Knee. 2014;21(5):911-5.

6. Beaupre LA, Menon MR, Almaazmi K, Kang SH, Dieleman S, Tsui B. Preoperative nerve blocks for hip fracture patients: A pilot randomized trial. Injury. 2021;52(3):548-53.

7. Chan EY, Fransen M, Sathappan S, Chua NH, Chan YH, Chua N. Comparing the analgesia effects of single-injection and continuous femoral nerve blocks with patient controlled analgesia after total knee arthroplasty. J Arthroplasty. 2013;28(4):608-13.

8. Chan EY, Teo YH, Assam PN, Fransen M. Functional discharge readiness and mobility following total knee arthroplasty for osteoarthritis: a comparison of analgesic techniques. Arthritis Care Res (Hoboken). 2014;66(11):1688-94.

9. Chaudet A, Bouhours G, Rineau E, Hamel JF, Leblanc D, Steiger V, et al. Impact of preoperative continuous femoral blockades on morphine consumption and morphine side effects in hip-fracture patients: A randomized, placebo-controlled study. Anaesth Crit Care Pain Med. 2016;35(1):37-43.

10. Cooke ME, Welch T, Gusakov O, Tornetta P, 3rd. Are Continuous Femoral Nerve Catheters Beneficial for Pain Management After Operative Fixation of Tibial Plateau Fractures? A Randomized Controlled Trial. J Orthop Trauma. 2019;33(12):e447-e51.

11. Dold AP, Murnaghan L, Xing J, Abdallah FW, Brull R, Whelan DB. Preoperative femoral nerve block in hip arthroscopic surgery: a retrospective review of 108 consecutive cases. Am J Sports Med. 2014;42(1):144-9.

12. Faunø P, Lund B, Christiansen SE, Gjøderum O, Lind M. Analgesic effect of hamstring block after anterior cruciate ligament reconstruction compared with placebo: a prospective randomized trial. Arthroscopy. 2015;31(1):63-8.

13. Gabriel RA, Kaye AD, Nagrebetsky A, Jones MR, Dutton RP, Urman RD. Utilization of Femoral Nerve Blocks for Total Knee Arthroplasty. J Arthroplasty. 2016;31(8):1680-5.

14. Guirro UB, Tambara EM, Munhoz FR. Femoral nerve block: Assessment of postoperative analgesia in arthroscopic anterior cruciate ligament reconstruction. Braz J Anesthesiol. 2013;63(6):483-91.

15. Hadzic A, Minkowitz HS, Melson TI, Berkowitz R, Uskova A, Ringold F, et al. Liposome Bupivacaine Femoral Nerve Block for Postsurgical Analgesia after Total Knee Arthroplasty. Anesthesiology. 2016;124(6):1372-83.

16. Hajian PH, N.; Nikouseresht, M.; Seif-Rabiee, M. A.; Yavarikia, A.;. Comparison of analgesic effect of femoral nerve block and continuous intravenous infusion pump, after anterior cruciate ligament reconstruction in first postoperative day. Anaesthesia, Pain & Intensive Care2019.

17. Helsø I, Jantzen C, Lauritzen JB, Jørgensen HL. Opioid Usage During Admission in Hip Fracture Patients-The Effect of the Continuous Femoral Nerve Block. Geriatr Orthop Surg Rehabil. 2016;7(4):197-201.

18. Kratz T, Dette F, Schmitt J, Wiesmann T, Wulf H, Zoremba M. Impact of regional femoral nerve block during general anesthesia for hip arthoplasty on blood pressure, heart rate and pain control: A randomized controlled study. Technol Health Care. 2015;23(3):313-22.

19. Krych A, Arutyunyan G, Kuzma S, Levy B, Dahm D, Stuart M. Adverse effect of femoral nerve blockade on quadriceps strength and function after ACL reconstruction. J Knee Surg. 2015;28(1):83-8.

20. Lomarat N, Akaraprasertkul J, Wongchompoo N, Boonsawek B, Sermsathanasawadi N. Ultrasound-guided femoral block in patients undergoing radiofrequency ablation of incompetent saphenous veins: A randomized controlled trial. Asian J Surg. 2023;46(1):174-9.

21. Lovald ST, Ong KL, Lau EC, Joshi GP, Kurtz SM, Malkani AL. Readmission and Complications for Catheter and Injection Femoral Nerve Block Administration After Total Knee Arthroplasty in the Medicare Population. J Arthroplasty. 2015;30(12):2076-81.

22. Magnussen RA, Pottkotter K, Stasi SD, Paterno MV, Wordeman SC, Schmitt LC, et al. Femoral Nerve Block after Anterior Cruciate Ligament Reconstruction. J Knee Surg. 2017;30(4):323-8.

23. Ogawa T, Seki K, Tachibana T, Hayashi H, Moross J, Kristensen MT, et al. Early recovery of basic mobility under femoral nerve block after hip fracture surgery - A propensity score matched pilot study. Injury. 2021;52(11):3382-7.

24. Peng L, Ren L, Qin P, Chen J, Feng P, Lin H, et al. Continuous Femoral Nerve Block versus Intravenous Patient Controlled Analgesia for Knee Mobility and Long-Term Pain in Patients Receiving Total Knee Replacement: A Randomized Controlled Trial. Evid Based Complement Alternat Med. 2014;2014:569107.

25. Polischuk MD, Kattar N, Rajesh A, Gergis T, King K, Sriselvakumar S, et al. Emergency Department Femoral Nerve Blocks and 1-Year Mortality in Fragility Hip Fractures. Geriatr Orthop Surg Rehabil. 2019;10:2151459319893894.

26. Ren YM, Tian MQ, Duan YH, Sun YB, Yang T, Hou WY, et al. Was femoral nerve block effective for pain control of medial opening-wedge high tibial osteotomy?: A single blinded randomized controlled study. Medicine (Baltimore). 2021;100(3):e23978.

27. Rowlands M, Walt GV, Bradley J, Mannings A, Armstrong S, Bedforth N, et al. Femoral Nerve Block Intervention in Neck of Femur Fracture (FINOF): a randomised controlled trial. BMJ Open. 2018;8(4):e019650.

28. Sahin L, Korkmaz HF, Sahin M, Atalan G. Ultrasound-guided single-injection femoral nerve block provides effective analgesia after total knee arthroplasty up to 48 hours. Agri. 2014;26(3):113-8.

29. Sengoku T, Nakase J, Morita Y, Asai K, Yoshimizu R, Kimura M, et al. Anterior cruciate ligament reconstruction with ultrasound-guided femoral nerve block does not adversely affect knee extensor strength beyond that seen with intravenous patient-controlled analgesia at 3 and 6 months postoperatively. Knee. 2022;34:252-8.

30. Singh AP, Kohli V, Bajwa SJ. Intravenous analgesia with opioids versus femoral nerve block with 0.2% ropivacaine as preemptive analgesic for fracture femur: A randomized comparative study. Anesth Essays Res. 2016;10(2):338-42.

31. Thomas K, Barrett B, Tupper R, Dacenko-Grawe L, Holm K. Pain management after total knee arthroplasty: a case–control study of continuous nerve block therapy. Orthop Nurs. 2014;33(5):268-76.

32. Unneby A, Svensson O, Gustafson Y, Olofsson B. Femoral nerve block in a representative sample of elderly people with hip fracture: A randomised controlled trial. Injury. 2017;48(7):1542-9.

33. Unneby A, Svensson PO, Gustafson PY, Lindgren APB, Bergström U, Olofsson PB. Complications with focus on delirium during hospital stay related to femoral nerve block compared to conventional pain management among patients with hip fracture - A randomised controlled trial. Injury. 2020;51(7):1634-41.

34. Uysal A, Altıparmak B, Yaşar E, Turan M, Canbek U, Yılmaz N, et al. The effects of early femoral nerve block intervention on preoperative pain management and incidence of postoperative delirium geriatric patients undergoing trochanteric femur fracture surgery: A randomized controlled trial. Ulus Travma Acil Cerrahi Derg. 2020;26(1):109-14.

35. Wiesmann T, Steinfeldt T, Wagner G, Wulf H, Schmitt J, Zoremba M. Supplemental single shot femoral nerve block for total hip arthroplasty: impact on early postoperative care, pain management and lung function. Minerva Anestesiol. 2014;80(1):48-57.

36. Wu JW, Wong YC. Elective unilateral total knee replacement using continuous femoral nerve blockade versus conventional patient-controlled analgesia: perioperative patient management based on a multidisciplinary pathway. Hong Kong Med J. 2014;20(1):45-51.

37. Xing JG, Abdallah FW, Brull R, Oldfield S, Dold A, Murnaghan ML, et al. Preoperative Femoral Nerve Block for Hip Arthroscopy: A Randomized, Triple-Masked Controlled Trial. Am J Sports Med. 2015;43(11):2680-7.

38. Yan SC, Fu SX, Li N, Mai L. Comparison of analgesic effects and postoperative cognitive function following total knee arthroplasty: continuous intravenous infusion of fentanyl vs. ultrasound-guided continuous femoral nerve block with ropivacaine. Am J Transl Res. 2021;13(4):3174-81.

39. Yao YY, Zhou QH, Yu LN, Yan M. Additional femoral nerve block analgesia does not reduce the chronic pain after total knee arthroplasty: A retrospective study in patients with knee osteoarthritis. Medicine (Baltimore). 2019;98(13):e14991.

40. Zhang J, Yuan Y, Zhang Y, Wang Y. Clinical effects of single femoral nerve block in combination with general anesthesia on geriatric patients receiving total knee arthroplasty. Pak J Med Sci. 2018;34(1):43-8.

41. Agarwala S, Bhadiyadra R, Menon A. Analgesic effectiveness of Local Infiltrative Analgesia alone versus combined single dose adductor canal block with Local Infiltrative Analgesia: A single centre case control study. Journal of clinical orthopaedics and trauma. 2020;11(Suppl 5):S717-S21.

42. Andersen HL, Gyrn J, Møller L, Christensen B, Zaric D. Continuous saphenous nerve block as supplement to single-dose local infiltration analgesia for postoperative pain management after total knee arthroplasty. Reg Anesth Pain Med. 2013;38(2):106-11.

43. Arumugam P, Ravi S, Ln S, Manickam P, Kanthan K. Evaluation of Analgesic Efficacy of Ultrasound-Guided Adductor Canal Block With 20 mL of 0.5% Ropivacaine in Patients Undergoing Knee Surgeries-A Randomized Control Trial. Asian J Anesthesiol. 2022;60(4):123-30.

44. Brush PL, Nanavati R, Toci GR, Conte E, Hornstein J. Surgeon-Performed Saphenous Nerve Block at the Medial Femoral Condyle for Arthroscopic Partial Meniscectomy and Meniscus Repair: A Randomized Control Trial. Cureus. 2022;14(7):e26971.

45. Espelund M, Fomsgaard JS, Haraszuk J, Dahl JB, Mathiesen O. The efficacy of adductor canal blockade after minor arthroscopic knee surgery--a randomised controlled trial. Acta Anaesthesiol Scand. 2014;58(3):273-80.

46. Espelund M, Fomsgaard JS, Haraszuk J, Mathiesen O, Dahl JB. Analgesic efficacy of ultrasound-guided adductor canal blockade after arthroscopic anterior cruciate ligament reconstruction: a randomised controlled trial. Eur J Anaesthesiol. 2013;30(7):422-8.

47. Gwam CU, Mistry JB, Richards IV, Patel D, Patel NG, Thomas M, et al. Does Addition of Adductor Canal Blockade to Multimodal Periarticular Analgesia Improve Discharge Status, Pain Levels, Opioid Use, and Length of Stay after Total Knee Arthroplasty? J Knee Surg. 2018;31(2):184-8.

48. Goytizolo EA, Lin Y, Kim DH, Ranawat AS, Westrich GH, Mayman DJ, et al. Addition of Adductor Canal Block to Periarticular Injection for Total Knee Replacement: A Randomized Trial. J Bone Joint Surg Am. 2019;101(9):812-20.

49. Gudmundsdottir S, Franklin JL. Continuous adductor canal block added to local infiltration analgesia (LIA) after total knee arthroplasty has no additional benefits on pain and ambulation on postoperative day 1 and 2 compared with LIA alone. Acta Orthop. 2017;88(5):537-42.

50. Hanson NA, Derby RE, Auyong DB, Salinas FV, Delucca C, Nagy R, et al. Ultrasound-guided adductor canal block for arthroscopic medial meniscectomy: a randomized, double-blind trial. Can J Anaesth. 2013;60(9):874-80.

51. Jæger P, Koscielniak-Nielsen ZJ, Schrøder HM, Mathiesen O, Henningsen MH, Lund J, et al. Adductor canal block for postoperative pain treatment after revision knee arthroplasty: a blinded, randomized, placebo-controlled study. PLoS One. 2014;9(11):e111951-e.

52. Johns N, Noye N, Wall C, Martin G, Loch A. Efficacy of Adductor Canal Blocks in Total Knee Arthroplasty. J Knee Surg. 2021.

53. Kejriwal R, Cooper J, Legg A, Stanley J, Rosenfeldt MP, Walsh SJ. Efficacy of the Adductor Canal Approach to Saphenous Nerve Block for Anterior Cruciate Ligament Reconstruction With Hamstring Autograft: A Randomized Controlled Trial. Orthop J Sports Med. 2018;6(10):2325967118800948.

54. Laksono RM, Isngadi I, Siswagama TA, Darmawan H, Asmoro AA, Basuki DR. Adductor canal block is superior to intravenous analgesia for multimodal postoperative pain management in anterior cruciate ligament reconstruction. Anaesthesia, Pain & Intensive Care. 2022;26(2):211-6.

55. Lan F, Shen Y, Ma Y, Cao G, Philips N, Zhang T, et al. Continuous Adductor Canal Block used for postoperative pain relief after medial Unicondylar Knee Arthroplasty: a randomized, double-blind, placebo-controlled trial. BMC Anesthesiol. 2019;19(1):114.

56. Leung P, Dickerson DM, Denduluri SK, Mohammed MK, Lu M, Anitescu M, et al. Postoperative continuous adductor canal block for total knee arthroplasty improves pain and functional recovery: A randomized controlled clinical trial. J Clin Anesth. 2018;49:46-52.

57. Nader A, Kendall MC, Manning DW, Beal M, Rahangdale R, Dekker R, et al. Single-Dose Adductor Canal Block With Local Infiltrative Analgesia Compared With Local Infiltrate Analgesia After Total Knee Arthroplasty: A Randomized, Double-Blind, Placebo-Controlled Trial. Reg Anesth Pain Med. 2016;41(6):678-84.

58. Nazemyanyazdi N, Delavari A, Saghafinia M, Emami MK. Comparison of Saphenous Nerve Block and Oral Oxycodone for Postoperative Pain Management in Total Knee Arthroplasty: A Randomized Clinical Trial. Trauma Monthly. 2021;26(5):265-72.

59. Nicolino TI, Costantini J, Carbó L. Complementary Saphenous Nerve Block to Intra-Articular Analgesia Reduces Pain After Total Knee Arthroplasty: A Prospective Randomized Controlled Trial. J Arthroplasty. 2020;35(6s):S168-s72.

60. Padki A, Vemula V, Purnomo G, Lim JBT, Liow LMH, Yeo SJ, et al. Adductor Canal Block Does not Confer Better Immediate Postoperative Pain Relief after Total Knee Arthroplasty. J Knee Surg. 2022.

61. Poon YY, Yang JC, Chou WY, Lu HF, Hung CT, Chin JC, et al. Is There an Optimal Timing of Adductor Canal Block for Total Knee Arthroplasty?-A Retrospective Cohort Study. J Pers Med. 2021;11(7).

62. Rames RD, Barrack TN, Barrack RL, Nunley RM. Effect of Adductor Canal Block on Acute Perioperative Pain and Function in Total Knee Arthroplasty. J Arthroplasty. 2019;34(7s):S164-s7.

63. Rousseau-Saine N, Williams SR, Girard F, Hébert LJ, Robin F, Duchesne L, et al. The Effect of Adductor Canal Block on Knee Extensor Muscle Strength 6 Weeks After Total Knee Arthroplasty: A Randomized, Controlled Trial. Anesth Analg. 2018;126(3):1019-27.

64. Sim JA, Lee MG, Jung WS, Lee BK, Lee BH. Clinical efficacy of adductor canal block in medial open wedge high tibial osteotomy. Knee. 2021;29:9-14.

65. Danisan G, Taydas O. Ultrasound-Guided Subgluteal Sciatic Nerve Block for Pain Management during Endovascular Treatment for Below-the-Knee Arterial Occlusions. J Vasc Interv Radiol. 2021.

66. Li Y, Zhang Q, Wang Y, Yin C, Guo J, Qin S, et al. Ultrasound-guided single popliteal sciatic nerve block is an effective postoperative analgesia strategy for calcaneal fracture: a randomized clinical trial. BMC Musculoskelet Disord. 2021;22(1):735.

67. Makkar JK, Bandyopadhay A, Jain K, Jafra A, Gopinathan NR, Singh P. Effect of perioperative sciatic nerve block on chronic pain in patients undergoing below-knee amputation: A randomised controlled trial. Indian J Anaesth. 2022;66(Suppl 6):S300-s6.

68. Schmitt HJ, Huberth S, Huber H, Münster T. Catheter-based distal sciatic nerve block in patients with Charcot-Marie-Tooth disease. BMC Anesthesiol. 2014;14:8.

69. Green C, Byrne AM, O'Loughlin P, Molony D, Harmon D, Masterson E. Surgeon delivered psoas compartment block in total hip arthroplasty. J Arthroplasty. 2014;29(2):393-6.
