# Supplementary material for: Peripheral regional anaesthesia and outcomes: a narrative review of the literature from 2013 to 2023
Source: Br J Anaesth. 2023 Nov 11;132(5):1082–96. doi: 10.1016/j.bja.2023.10.013 (PMC11103102; doi:10.1016/j.bja.2023.10.013)
Supplement: Multimedia component 4 [file mmc4.docx]

**Cervical nerve block**

| **Author** | **Design** | **N** | **Surgery** | **Technique** | **Anaesthetics** | **Primary outcome** | **Result primary outcome** | **Success rate** | **Pain** | **Analgesia** | **PONV or other side effects** | **Long-term outcomes** | **Other (quality of recovery, satisfaction, functional recovery, hospital stay)** | **Complications** |
| --- | --- | --- | --- | --- | --- | --- | --- | --- | --- | --- | --- | --- | --- | --- |
| Aweke et al.^1^ | RCT | 66 | Thyroidectomy | Landmark technique – single shot | 33 CB + GA vs. GA |  |  |  | - Lower NRS at PACU (median [IQR] 3 [2-4] vs. 5 [3-6]), at 3 hr (2 [1-3] vs. 4 [3-5]), all p < 0.05).  - Also lower at 6, 12 and 24 hr*  - Higher proportion of patients NRS > 4 during 24 hours (10.6% vs. 30.3%, p = 0.003).  ** No values mentioned* | - Lower total IV tramadol use 24 hr (median [IQR] 0 [0-50] IV mg vs. 100 [25-150], p = 0.001)  - No dif. in IO tramadol use  - Longer time to first rescue analgesic (median [IQR] 360 [190-270] min vs. 180 [65-3360], p = 0.006)  - No dif. in 24 hr IM diclofenac use | No dif. in PONV |  |  |  |
| Camerani et al.^2^ | RCT | 64 | Carotid endarterectomy | Landmark – single shot | 32 CB + GA vs. GA | Aldrete score at 5 minutes | Higher (= better, median [IQR] 8.5 [8-9] vs. 7 [6-8], p = 0.001) |  | No dif. in pain at 0, 5, 10, 30 min | - Less IO remifentanil (mean (SD) 1107 (357) vs. 1737 (713) mcg, p < 0.001).  - No dif. in IO propofol | Less PONV at 0 min (6.3% vs. 62.5%), 5 min (9.4% vs. 56.3%), 10 min (0% vs. 84.4%), and 30 min (0% vs. 34.4$), all p < 0.001) |  |  | None related to block |
| Chen et al.^3^ | RCT | 96 | Thyroidectomy | ? – single shot | 48 CB + GA vs. GA |  |  |  | Lower VAS at 12 hr (mean (SD) 5.1 (0.2) vs. 6.5 (0.3)), 24 hr (6.1 (0.4) vs. 7.3 (1)), and 48 hr (1.1 (0.2) vs. 2.6 (0.5)), all p < 0.01. No dif. at 4 and 8 hr. |  | Less adverse reactions (4 (8.3% vs. 11 (22.9%), p < 0.05) |  |  |  |
| Deepika et al.^4^ | RCT | 30 | Radical mastoidectomy | US – single shot | 15 CB + GA vs. GA | VAS | - Lower VAS at rest at 1 hr (mean (SD) 0.2 (0.6) vs. 1.9 (2.1), p = 0.012). No dif. at 5, 10, and 15 minutes or 4, 8, 12, 18, 24 hours  - Lower VAS on movement at 1 hr (mean (SD) 0.6 (0.9) vs. 2.7 (2.4)), 8 hr (0.3 (0.5) vs. 1.3 (1.7)), 12 hr (0 (0) vs. 0.7 (0.9)), all p < 0.05. No dif. at 5, 10, and 15 minutes or 4, 8, 18, 24 hours |  |  | - Less diclofenac use at 1 hr (mean (SD) 0 (0) mg vs. 0.3 (0.5) mg, p = 0.041) |  |  |  | None related to block |
| Gong et al.^5^ | Prospective comparative | 48 | Total parathyroidectomy with autotransplantation | US – single shot | 24 CB + GA vs. GA | VAS | Lower at 1, 3, 10, 24, 48 hr (p < 0.05). No dif. at 10 min.  * *No values shown* |  | Fewer incidences of pharyngeal pain (0 vs. 8 (33%), p = 0.002) | - Lower IO remifentanil use (mean (SD) 2.6 (0.9) mg vs. 3.4 (0.8) mg, p = 0.002)  - No dif. in postoperative sufentanil use  - Less propofol (mean (SD) 659 (260) vs. 1112 (278) mg, p < 0.001  - Less cisatracurium (mean (SD) 14.6 (5.3) vs. 21.2 (5.8), p < 0.001) | - No dif. in PONV  - No dif. in dyspnoea |  | - Shorter extubation time (mean (SD) 5.8 (2.1) min vs. 11.4 (3), p < 0.001) | None related to block |
| Goulart et al.^6^ | RCT | 100 | Total thyroidectomy | Landmark technique – single shot | 50 CB + GA vs. GA |  |  |  | - Fewer patients reporting VAS pain score > 3 at PACU 15 min (3 (6%) vs 28 (56%), at 30 min (1 (2%) vs. 17 (34%)), both p < 0.001). No dif. at arrival, 45 min, 60 min  - No dif. in VAS score at ward | - Fewer patients use nalbuphine at PACU 15 min (3 (6%) vs. 28 (56%), 30 min (1 (2%) vs. 17 (34%)), both p < 0.001). No dif. at arrival, 45, 60 min  - Fewer patients use nalbuphine at ward 4 hr (3 (6%) vs. 10 (20%), p = 0.037). No dif. at 0, 8, 12 hr | - Less PONV at 45 min at PACU (16% vs. 0%, p = 0.006), no dif. at arrival, 15, 30, 60 min.  - Fewer incidences of PONV at ward 4 hr (0 vs. 9 (18%), 8 hr (0 vs. 7 (14%)) both, p < 0.05). No dif. at arrival, 12 hr. |  |  | None related to block |
| Gürkan et al.^7^ | RCT | 49 | Thyroid surgery | US – single shot | 25 CB + GA vs. GA | Postoperative opioid consumption | Lower at 6 hr (median [IQR] 4 [1-5] IV mg vs. 5 [4-7.5]), 12 hr (8 [2.5-9.5] vs. 9 [6-16]), 24 hr (9 [3.5-14.5] vs. 11 [8-22.5], p < 0.05). No dif. at 1 hr. |  | No dif. in VAS score at 1, 6, 12, 24 hour |  |  |  |  | 7 patients hoarseness following block, in all patients relieved within 6 hours |
| Hu et al.^8^ | RCT | 82 | Uraemia patients undergoing total parathyroidectomy | US – single shot | 41 CB + GA vs. sham + GA | QoR-40 | Higher (= better, median [IQR] 162 [157.5-166.5] vs. 149 [144-153.5], p < 0.001) |  | - Lower VAS score at 2, 4, 8, 12, 24 hr after surgery (p < 0.001)  * *No values or dispersion measures dislplayed* | - Lower IO remifentanil use (mean (SD) 533.4 (59.9) μg vs. 580.1 (76.1), p < 0.001)  - Longer time to first rescue analgesia (mean 15.4 (1.9) hr vs. 8.3 (1.2), p = 0.018)  - Fewer patients requiring rescue analgesia (20 (48.8%) vs. 36 (87.8%), p < 0.001)  - Lower tramadol use (mean (SD) 24.4 (25.3) IV mg vs. 43.9 (16.6), p < 0.001) | Lower incidence of PONV (2 (4.9%) vs. 10 (24.4%), p = 0.013) |  |  | None related to block |
| Kale et al.^9^ | RCT | 60 | Thyroid | Landmark – single shot | 20 preCB + GA vs.  20 postCB + GA vs. 20 GA |  |  |  | Lower VAS at rest during first 48 hr in both preCB and postCB (mean 2.3 vs. 2.7 vs. 4.2, p < 0.05), at neck movement (mean 2.1 vs. 2.1 vs. 5.0, p < 0.001), at swallowing (mean 2.3 vs. 2.3 vs. 4.6, p < 0.001), and at vocalisation (mean 2.2 vs. 2.1 vs. 4.9, p < 0.001).  ** No dispersion measures shown* | - Lower IO fentanyl us in group preCB vs. postCB and GA (mean (SD) 103 (8) μg vs 143.5 (10) vs. 138.3 (11), p < 0.05)  - Longer time until first rescue analgesic request both in both preCB and postCB (mean 4 hr vs. 6.3 vs 1, p < 0.001)*  * *No dispersion measures shown* | No dif. in PONV |  |  | None related to block |
| Kannan et al.^10^ | RCT | 50 | Thyroid | US – single shot | 25 CB + GA vs. sham + GA |  |  |  | Lower VAS at 30 min (median 0 vs. 2), 60 min (0 vs. 4) and 2 hr (1 vs. 4), all p < 0.001)  ** No dispersion values shown* | - Lower mean sevoflurane concentration at 30, 60, min (p < 0.05), no dif. at 90 min  - No dif. in IO fentanyl or propofol use |  |  |  | One patient had hoarse voice, recovered after 12 hr |
| Majdoub et al.^11^ | RCT | 60 | Thyroidectomy | US – single shot | 29 CB + GA vs. GA |  |  |  | Lower VAS at 0 hr (median [IQR] 4 [3-5] vs. 5 [4-7], p = 0.005), 12 hr (3 [2-3] vs. 3 [3-4], p = 0.0059), 24 hr (3 [2-3] vs. 3 [3-4], p = 0.008). No dif. at 1, 2, 6 hr. | - Lower IO remifentail (mean (SD) 828 (192) vs. 1350 (494) mcg, p < 0.001).  - Lower morphine use after surgery (mean 2.3 (3.1) IV mg vs. 4.1 (3.5), p = 0.037) | No dif. in PONV |  | No dif. in satisfaction |  |
| Mariappan et al.^12^ | RCT | 46 | Anterior cervical discectomy and fusion | Landmark technique – Single shot | 23 CB + GA vs. GA | QoR-40 at 24 hr | Higher (= better, median [IQR] 179 [116-195] vs. 157 [97-196], p = 0.002) |  | No dif. in VAS scores (up to 12 hr) | No dif. in opioid consumption at 24 hr | - No dif. in incidence of adverse effects in first 2 hr  - Less N/V & dysphagia at 24 hr.  ** No values shown* | No dif. in NDI score at three months |  | Five patients had hoarseness (2 CB, 3 no-CB) |
| Ozgun et al.^13^ | RCT | 60 | Thyroidectomy | Landmark – single shot | 30 CB + GA vs. GA | Postoperative analgesic consumption | Lower PCA-tramadol consumption at 24 hr (mean 164 ± 96 vs. 261 ± 109, p = 0.001)  * *Unclear if mg or ml* |  | - Lower NRS at PACU (mean (SD) 2 (1.6) vs. 3.3 (2.2), p = 0.013). No dif. at 1, 2, 6, 12, 24 hr.  - Less patients pain score ≥ 6 (10% vs. 33%, p = 0.02) | - Less patients requiring rescue analgesics (4 vs. 11, p = 0.03)  - No dif. in IO fentanyl requirement |  |  |  | Two patients had postoperative subcutaneous emphysema in CB group, regressed at the 12^th^ hour. |
| Waheb et al.^14^ | RCT | 90 | Tympanomastoid | US – single shot | 45 CB + GA vs. GA | IO fentanyl consumption | Less (mean (SD) 162.2 (26.5) mcg vs. 181.1 (28.8), p = 0.002) |  | No dif. in VAS | No dif. in need for ketolac and pethidine postoperative | Less PONV between 0 – 8 hr after surgery, p < 0.05. No dif. after 8 hr,  ** No incidences/ or percentages shown.* |  |  | None related to block |
| Yao et al.^15^ | RCT | 71 | Thyroid cancer | US – single shot | 36 CB + GA vs. sham + GA | QoR-15 at 24 hr | Higher (median [IQR] 118 [115-120] vs. 110 [106-112], p < 0.001) |  | Lower AUC VAS first 24 hours, p < 0.001). | - Longer time to first rescue analgesia (median [IQR] 18.8 [17-21] hr vs. 8.1 [5.6-10.5], p <0,001)  - Fewer patients requiring rescue analgesia (17/36 vs. 29/35, p = 0.002)  - Lower cumulative morphine use 24 h (median [IQR] 0 [0-2] IV mg vs. 2 [2-4], P < 0,001)  ** Unclear which centre and dispersion measures used for morphine* | Reduced PONV (1 (2.8%) vs. 7 (20%), p = 0.028) |  | - Shorter LOS PACU (median [IQR] 20 [18-20 min vs. 24 [20-28], p < 0.001)  - Higher patient satisfaction (median [IQR] 9 [8-10] vs. 8 [7-9], p = 0.024) | None related to block |
| Zeng et al.^16^ | RCT | 106 | Craniotomy | US – single shot | 53 CB + GA vs. sham + GA | Cumulative PCA-sufentanil consumption at 24 hours | Lower (mean (SD) 5.0 (3.8) mcg vs. 9.8 (9.3), p < 0.001) |  | - Lower NRS-AUC at rest 48 hr (median [IQR] 95 [68-178] vs. 155 [116-253], p < 0.01.  - Lower NRS-AUC at movement 48 hr (164 [115-218] vs. 233 [155-283], p < 0.01.  - Lower incidence of severe pain within 24 hr (7.5% vs. 26.4%, p = 0.01) | - Lower total sufentanil (mean (SD) 45.0 (11.5) mcg vs. 54.5 (16.1), p < 0.01) | - No dif. in PONV  - No dif. in Ramsay score  - No dif. in dizziness |  | - No dif. in satisfaction  - No dif. in LOS  - No dif. in Sleep Quality Score |  |

**Abbreviations:** N = number of patients, PONV = perioperative nausea and vomiting, RCT = randomised controlled trial, CB = cervical nerve block, GA = general anaesthesia, NRS = numeric rating scale, PACU = postoperative anaesthesia care unit, IV = intravenous, IO = intraoperative, dif. = difference, IM = intramuscular, VAS = visual analogue scale, US = ultrasound, QoR = quality of recovery, N/V = nausea/vomiting, NDI = neck disability index, PCA = patient-controlled analgesia, AUC = area under the curve, LOS = length of stay

**Intercostal nerve block**

| **Author** | **Design** | **N** | **Surgery** | **Technique** | **Anaesthetics** | **Primary outcome** | **Result primary outcome** | **Success rate** | **Pain** | **Analgesia** | **PONV or other side effects** | **Long-term outcomes** | **Other (quality of recovery, satisfaction, functional recovery, hospital stay)** | **Complications** |
| --- | --- | --- | --- | --- | --- | --- | --- | --- | --- | --- | --- | --- | --- | --- |
| Abadi et al.^17^ | Retrospective | 106 | Sternotomy - CABG | ?– ?  * *Unclear which technique was used* | 53 INB + ? vs. ?  * *Unclear which anaesthetic was used* |  |  |  | - Lower maximum pain score first 24 hr (mean [range] 6.2 [0-10] vs. 7.7 [4-10],  p = 0.015)  - No dif. in average pain score | Lower total amount of opioid 72 hr (32 (33) vs. 150 (95) oral MME, p < 0.01) |  |  | No dif. in LOS |  |
| Ahmed et al.^18^ | RCT | 60 | Diagnostic VATS | Landmark – Single shot | 30 INB + GA vs. GA | 24 hr morphine requirement | No dif. |  | Lower VAS at 1 hr (mean 3.0 vs. 4.1), 6 hr (2.6 vs. 3.6), 24 hr (1.8 vs. 2.5, all p < 0.05). No dif. at 12 hr.  ** No dispersion values mentioned* | Less PCA-morphine 0-6 hr (mean (SD) 12.7 (7.1) mg IV vs. 17.3 (8.4), p < 0.05). No dif. between 6-24 hr. | No dif. in PONV |  |  | None related to block |
| Chen et al.^19^ | RCT | 41 | Sternotomy - mediastinal mass resection | US – single shot | 20 INB + GA vs. sham + GA | Cumulative sufentanil use 24 hr after surgery | Lower (mean (SD) 54.1 (11.1) μg vs. 67.7 (8.9), p < 0.001) |  | Lower NRS at rest and coughing at 1, 3, 6, 12, 24 hr (p < 0.05).  **No values given* | No dif. in patients requiring rescue analgesics within 24 hr | - No dif. in PONV  - No dif. in dizziness, excessive sedation and respiratory depressions |  | Higher satisfaction score (mean (SD) 8.2 (1.0) vs. 6.7 (0.6), p < 0.001) |  |
| Honey et al.^20^ | RCT | 63 | Percutaneous nephrolithotomy | Landmark + fluoroscopy – single shot  * *Unclear which technique was used* | 30 INB + ? vs. ? + sham  * *Unclear which anaesthetic was used* | Narcotic use during hospital stay | No dif. |  |  | Lower narcotic use first 3 hr (mean (SD) 2.4 (3.1) MME vs. 4.3 (3.8), p = 0.034). No dif. after 3 hr  **Unclear whether IV or oral MME* |  |  | - No dif. in LOS  - Durable improvement postoperative HRQL score (p = 0.034)  **No values given* | None related to block |
| Hsieh et al.^21^ | Retrospective | 78 | VATS anatomic resections | Endoscopic - continuous | 39 INB + GA vs. GA |  |  |  | Lower NRS at POD 0 (mean (SD) 2.9 (1.8) vs. 3.8 (1.6)), POD 1 (1.5 (1.1) vs. 2.9 (1.9), both p < 0.05).  No dif. at POD2, POD3, discharge day | Lower morphine use (mean (SD) 2.6 (3.6) IV mg vs. 5.5 (6.4), p = 0.017) |  |  | - Better triflow performance at POD 1 (mean 2.3 vs. 1.9), POD 2 (2.6 vs. 2.3), both p < 0.05. No dif. at other times.*  - Shorter chest tube drainage duration (mean 3.2 days vs. 5.0, p = 0.001).*  - Shorter LOS (mean 4.2 ± 1.9 days vs. 6.3 ± 4.0, p = 0.005).  ** No dispersion measures shown* |  |
| Kang et al.^22^ | Prospctive | 44 | Augmentation mammoplasty | Landmark – single shot | 34 INB + GA vs. GA |  |  |  | Lower VAS at PACU (mean (SD) 3.5 (1.8) vs. 7.1 (0.7)), 30 min (3.0 (1.5) vs. 7 (0.7)), 60 min (2.7 (1.5) vs. 5.5 (0.7)), 120 min (2.0 (1.4) vs. 4.6 (0.8), p < 0.001). |  |  |  | Shorter PACU discharge time (mean (SD) 189.7 (15.2) min vs. 489 (172.2), p < 0.001) | None related to block |
| Kang et al.^23^ | Retrospective | 40 | VATS - lobectomy | Thoracoscopic – single shot | 20 INB + GA vs. GA |  |  |  | Lower NRS at 1 hr (mean (SD) 3.5 (0.8) vs. 5.6 (1.2), p < 0.01), at 12 hr (mean 3.5 (1.4) vs. 4.7 (1.5), p = 0.014). No dif. at 24 hr. | Less NSAID injections until chest tube removal (mean (SD) 1 (1.4) vs. 2.4 (2.5), p = 0.038) |  |  |  |  |
| Kang et al.^24^ | Retrospective | 40 | VATS – wedge resection | Thoracoscopic – single shot | 20 INB + GA vs. GA | NRS measured at multiple time intervals |  |  | Lower NRS at 1 hr (mean (SD) 4.1 (1.5) vs. 5.6 (1.3), p = 0.001), at 12 hr (3.8 (1.7) vs. 5 (1.4), p = 0.022. No dif. at 24 hr. | Less opioid injections until chest tube removal (mean 0.4 (0.6) vs. 1.2 (1.3), p = 0.025) |  |  | No dif. in LOS |  |
| Lee et al.^25^ | RCT | 79 | Sternotomy - CABG | Under direct vision of surgeon – single shot | 38 INB + GA vs. sham + GA | NRS measured at multiple time intervals | No dif. in NRS when measured individually (1, 2, 4, 8, 12, 24, 36, 48, 60, 72 hr). However, using linear mixed effects model lower overall pain levels (coefficient estimate was -0.54, p = 0.040)  * *No values mentioned* |  |  | Lower MME at 2 hr (median 0 vs 5.0 mg, p = 0.047). No dif. at 1, 2, 4, 8, 12, 24, 36, 48, 60, 72 hr, nor in overall time.  **Unclear whether IV or oral MME*  ** No dispersion values shown* |  |  | No dif. in extubation time, length of stay, ICU length of stay, return to normal activities/work | None related to block |
| Lewis et al.^26^ | Retrospective | 64 | Thoracotomy - lung transplantation | Surgeon performed – 3-level single shot | 44 INB + GA vs. GA |  |  |  | No dif. in pain scores POD 1, 2, 3 or 4 | - Lower total opioid consumption POD 1-4 (mean 228 mg vs. 517, p = 0.32)  - No dif. in opioids at discharge home  * *No dispersion values* |  |  | No dif. in LOS |  |
| Ozkan et al.^27^ | RCT | 40 | Percutaneous nephrolithotomy | US + NS – single shot | 20 INB + GA vs. sham + GA |  |  | 100% succes | - Lower VAS at rest 10 min (median [IQR] 2 [1-3] vs. 4 [3.5-7]), 20 min (2.5 [2-3] vs. 5 [4-5.7]), 1 hr (2 [2-3] vs. 4.5 [3-6]), 4 hr (2 [1-3] vs. 4.5 [4-5]), 8 hr (2 [0.5-3] vs. 4 [3-4.7]), 12 hr (2 [1-2.7] vs. 3.5 [3-5]), 24 hr (0.5 [0-2] vs. 3 [2-3.7]), all p < 0.05  - Lower VAS during movement at all mentioned time points | Lower total tramadol use (mean (SD) 97.5 (39.5) IV mg vs. 199.7 (77.6), p < 0.05) | Nausea score > 2 (0 vs. 7 (35%), p = 0.04 |  | - Higher patient satisfaction (mean 8.5 (1.7) vs. 2.8 (0.6), p < 0.05).  - No dif. in PACU discharge time | None related to block |
| Ranganathan et al.^28^ | RCT | 60 | Thoracotomy - lung resection | Under direct vision of surgeon – single shot | 30 INB + GA epidural vs. GA + epidural | Pain scores 2 to 4 hr after surgery | No dif. |  | No dif. at pain 18 to 24 hr at rest or coughing. | No dif. in fentanyl consumption after surgery |  |  |  |  |
| Shah et al.^29^ | Retrospective | 132 | Breast reconstruction (2 groups; bilateral and unilateral) | Direct vision – single shot | 89 INB + GA vs. GA |  |  |  |  | - Lower morphine use bilateral (5.2 IV mg vs. 12.7, p = 0.041 and unilateral (2.8 mg vs. 8.2 mg, p = 0.007)  - No dif. in oral oxycodone use in bilateral and unilateral  ** Unclear which centre measures are used, no dispersion measures shown* | No dif. in antiemetic’s use |  | - Shorter LOS in bilateral (1.9 days vs. 2.3, p = 0.001) No dif. in unilateral  - Expected hospital billing cost without INB $28,710.33 vs. with INB $ 25,837.19  ** Unclear which centre measures are used, no dispersion measures shown* | 1 pneumothorax in patient who had simultaneous port placement which resolved without intervention |
| Vilvanathan et al.^30^ | RCT | 90 | Sternotomy - CABG | US – single shot | 45 INB + GA vs. GA | NRS pain score at rest and coughing | Significantly less at rest at 15 min (mean 3.3 vs. 4.3), at 4 hr (2.9 vs. 4.0), 8 hr (2.6 vs. 4.2), 12 hr (2.6 vs. 4.5), 16 hr (2.4 vs. 4.4), 20 hr (2.7 vs. 4.6), 24 hr (3.5 vs. 5.1).  Significantly less during breathing exercise at 4 hr (3.0 vs. 4.5), 8 hr (3.2 vs. 5.1), 12 hr (3.4 vs. 5.1), 16 hr (3.3 vs. 5.3), 20 hr (3.5 vs. 5.4), 24 hr (3.7 vs. 5.5).  ** No dispersion values, no p-values shown.* |  |  | - Lower IO fentanyl use (mean (SD) 675.6 (188.8) mg vs. 976.7 (169.1), p < 0.0001)  -Fewer patients required rescue analgesia at 15 min (0 vs. 4), 4 hr (1 vs. 8 ), 8 hr (2 vs. 10), 12 hr (2 vs. 8), 16 hr (1 vs. 9), 20 hr (2 vs. 8) all p < 0.05) | -Less nausea (2 (4.4%) vs. 10 (22.2%), p < 0.05)  - Less vomiting (1 (2.2%) vs. 8 (17.7%), p < 0.05)  - Less pruritis (2 (6.7% vs. 13 (28.8%), p = 0.006)  - Less excessive sedation (Ramsay sedation scale > 3, 2 (4.4% vs. 11 (24.4%), p = 0.007) |  |  |  |
| Wang et al.^31^ | RCT | 100 | Thoracotomy - oesophageal cancer | ? –Single shot | 50 INB + GA vs. GA |  |  |  | Lower VAS at 2, 12, 24 hr (p < 0.05)  * *No values shown* |  |  |  | Higher MMSE score at 2 hr, 12 hr, 24 hr (p < 0.05).  * *No values shown* |  |
| Wu et al.^32^ | Retrospective + PSM | 185  *PSM:*  100 | VATS | Direct vision - continue infusion | 50 INB + GA vs. GA |  |  |  | *After PSM*  - Lower NRS at POD 0 (mean (SD) 2.1 (1.7) vs. 4.0 (2.0)), POD 1 (1.5 (1.0) vs. 2.6 (1.0)), POD 2 (1.1 (0.9) vs. 1.6 (1.1)), POD 3 (0.7 (0.8) vs. 1.23 (0.9), all p < 0.05)  - No dif. in discharge pain score | *After PSM*  Lower morphine use dosage (mean (SD) 3.0 (3.9) IV mg vs. 7.14 (8.5), p = 0.003) |  |  | Shorter hospital stay (mean (SD) 4.3 (1.8) days vs. 5.5 (3.5), p = 0.042 | None related to block |
| Xu et al.^33^ | RCT | 95 | VATS - lung cancer | Thoracoscopic visualization – single shot | 50 INB + GA vs. GA |  |  |  | Lower VAS at 2 hr (mean (SD) 1.8 (0.5) vs. 3.8 (0.9)), 4 hr (2.4 (0.7) vs. 4.6 (1.2)), 8 hr (2.9 (0.9) vs. 5.2 (1.5)), 12 hr (3.6 (1.2) vs. 5.7 (1.4)), 24 h (3.3 (0.9) vs. 5.5 (1.3)), all p < 0.05 | Fewer PCA compressions (mean (SD) 14.4 (4.2) vs. 28.0 (8.5), p < 0.001) | No dif. Ramsay sedation scores or N/V |  | Lower (= better) satisfaction score (mean (SD) 1.9 (0.6) vs. 3.0 (0.9) p < 0.001) |  |
| Zhan et al.^34^ | RCT | 30 | Minimally invasive mitral valve surgery | Single shot T3-T7 + catheter posterior axillary line  * *Unclear which technique was used* | 15 INB + GA vs. GA |  |  |  | Lower VAS score 24 hr (mean (SD) 3.7 (0.6) vs. 5.3 (0.6), p < 0.001) | Less IO sufentanil use (mean 7.7 (0.4) μg/kg vs. 9.4 (0.5), p < 0.001) |  |  | -Shorter ICU stay time (mean 29 (1.4) hr vs. 30.5 (1.1), p < 0.01)  -Shorter extubation time (mean 17.7 (1.2) hr vs. 20.6 (1.1), p < 0.001) | None related to block |
| Zhu et al.^35^ | RCT | 81 | Thoracotomy - esophagotomy | US – single shot | 40 INB + GA vs. GA |  |  |  | Lower VAS at rest and on cough at 1, 2 & 4 hr (p < 0.01)  ** No values shown* | Lower PCA-sufentanil consumption in 48 hr (156.2 (9.1) μg vs. 183.1 (11.3), p < 0.001) | Lower incidence of PONV (5 (12.5%) vs. 13 (31.7%), p = 0.038) |  | Shorter PACU stay (mean (SD) 25.2 (8.3) min vs. 35.8 (10.4), p < 0.001) | None related to block |

**Abbreviations:** N = number of patients, PONV = perioperative nausea and vomiting, CABG = coronary artery bypass grafting, INB = intercostal nerve block, dif. = difference, MME = milligram morphine equivalent, LOS = length of stay, RCT = randomised controlled trial, VATS = video assisted thoracoscopic surgery, GA = general anaesthesia, VAS = visual analogue scale, PCA = patient controlled analgesia, IV = intravenous, US = ultrasound, NRS = numeric rating scale, HRQL = health-related quality of life, POD = postoperative day, PACU = postoperative anaesthesia care unit, NSAID = non-steroidal anti-inflammatory drugs, ICU = intensive care unit, IO = intraoperative, MMSE = mini mental state examination, N/V = nausea/vomiting

**Ilioinguinal/ilihypogastric**

| **Author** | **Design** | **N** | **Surgery** | **Technique** | **Anaesthetics** | **Primary outcome** | **Result primary outcome** | **Success rate** | **Pain** | **Analgesia** | **PONV or other side effects** | **Long-term outcomes** | **Other (quality of recovery, satisfaction, functional recovery, hospital stay)** | **Complications** |
| --- | --- | --- | --- | --- | --- | --- | --- | --- | --- | --- | --- | --- | --- | --- |
| Elahwal et al.^36^ | RCT | 64 | Caesarean section | US – single shot | 32 IINB + IHNB + spinal vs. spinal | IV morphine consumption | Less (mean (SD) 4.5 (1.5) vs. 8.9 (2) mg, p < 0.001) |  | - Lower VAS at rest at 2, 4, 6, 12 hr after surgery, p < 0.05. Not 30 min and 24 hr.  - No dif. in VAS during movement * *No values mentioned* | - Longer time to first rescue analgesia (mean (SD) 12.3 (9.9) vs. 3.8 (1) hr, p < 0.001)  - Less patients needed rescue analgesia (19 (59%) vs. 32 (100%), p < 0.001. | - Less nausea (22 (7%) vs. 72 (23%), p < 0.001) | - Lower incidence of chronic postsurgical pain at three months (10 (3%) vs. 33 (10%), p = 0.028) and at six months (1 (3.3%) vs. 6 (20%), p = 0.044.  -Lower severity of chronic postsurgical pain at three months (mean (SD) 0.09 (0) vs. 0.8 (0.5), p = 0.001), and at six months (mean 0.03 (0.2) vs. 0.2 (0.4), p = 0.014) |  |  |
| Gu et al.^37^ | RCT | 62 | Cervical cancer | US- single shot | 31 IINB + IHNB + GA vs. GA |  |  |  | Lower VAS postoperative (mean (SD) 3.4 (0.6) vs. 4.9 (0.7), p = 0.023) | - Less presses PCA-sufentanil (mean (SD) 6.2 (1.3) vs. 9.7 (1.6), p = 0.018)  - Less IO propofol (mean (SD) 11.2 (3.4) vs. 18.4 (5.3) mg, p = 0.027).  - Less IO sufentanil (mean 15.4 (3.2) vs. 21.7 (4.6) mcg, p = 0.026). | Less adverse events (3 (10%) vs. 11 (35%), p < 0.01) |  |  |  |
| Naghshineh et al.^38^ | RCT | 80 | Caesarean section | Direct vision – single shot | IINB + GA vs. GA |  |  |  | - Lower VAS at rest 0 hr (mean (SD) 1.1 (0.3) vs. 3 (1.2)), 1 hr (1.1 (0.3) vs. 2.3 (0.4)) 2 hr (1.2 (0.4) vs. 2.6 (0.9)), 4 hr (1.5 (0.5) vs. 2.5 (1)), 12 hr (2.1 (1.1) vs. 3 (1.2)), p < 0.005). No dif. at 6, 24 hr  - Lower VAS in sitting position hr (mean (SD) 1.8 (0.4) vs. 2.4 (0.6)), 2 hr (1.8 (0.4) vs. 2.9 (1.3)), 4 hr (1.9 (0.4) vs. 2.9 (1.2)) 12 hr (2.6 (1.1) vs. 3.6 (1.5)), 24 hr (2 (0.5) vs. 1.8 (0.4), p < 0.005). No dif. at 6 hr  - Lower VAS when walking at 12 hr (mean 2.9 (1.5) vs. 4.1 (1.8), p = 0.002). No dif. at 24 hr | - Less patients using narcotics at 0 hr (0 vs. 12 (37.5%), 2 hr (0 vs. 7 (17.5%), 4 hr (0 vs. 11 (27.5%), 12 hr (10 (25%) vs. 22(55%), all p < 0.05. No dif. at 6, 24 hr  - Longer time to first time receiving narcotic (mean (SD) 7.7 (2.0) hr vs. 3.4 (2.8), p < 0.001) | - No dif. in vomiting  - No dif. in anti-emetic use |  |  |  |
| Raoofi et al.^39^ | RCT | 150 | Caesarean section | Landmark + direct vision – single shot 2 hr after surgery (through catheters placed IO) | IINB + IHNB + spinal vs. sham + spinal |  |  |  | - Lower VAS at 4 hr (mean (SD) 1.2 (0.9) vs. 1.9 (0.9)), 6 hr (0.5 (0.6) vs. 1.2 (0.5)), p < 0.001). No dif. at 2, 8 hr | Fewer patients receiving pentazocine (n = 4 (9.3%) vs. 55 (69.4%), p < 0.001) | - No dif. in PONV  - No dif. in itching |  |  | One case of haematoma at catheter site |

**Abbreviations:** N = number of patients, PONV = perioperative nausea and vomiting, RCT = randomised controlled trial, US = ultrasound, IINB = ilioinguinal nerve block, IHNB = iliohypogastric nerve block, IV = intravenous, VAS = visual analogue scale, dif. = difference, GA = general anaesthesia, PCA = patient-controlled analgesia, IO = intraoperative

**References**

1. Aweke Z, Sahile WA, Abiy S, Ayalew N, Kassa AA. Effectiveness of Bilateral Superficial Cervical Plexus Block as Part of Postoperative Analgesia for Patients Undergoing Thyroidectomy in Empress Zewditu Memorial Hospital, Addis Ababa, Ethiopia. Anesthesiology Research and Practice. 2018;2018.

2. Camerani S, Capuzzo M, Ieffa E, Pescolderung M, Braccini L, Volta CA. Total intravenous anesthesia with superficial cervical block or morphine transition in patients undergoing carotid endarterectomy. Minerva Anestesiol. 2014;80(6):676-84.

3. Xiao Chen YQ, Xing Zhao, Zhihu Liu, Zhenhua Qu. Effect of a combination of general anesthesia and superficial cervical plexus block with ropivacaine on patients undergoing thyroidectomy. Tropical Journal of Pharmaceutical Research2022.

4. Deepika V, Ahuja V, Thapa D, Gombar S, Gupta N. Evaluation of analgesic efficacy of superficial cervical plexus block in patients undergoing modified radical mastoidectomy: A randomised controlled trial. Indian J Anaesth. 2021;65:S115-s20.

5. Gong J, Yao Y, Wang Y. Effects of ultrasound-guided bilateral cervical plexus block combined with general anesthesia in patients undergoing total parathyroidectomy and partial gland autotransplantation surgery. Local and Regional Anesthesia. 2021;14:75-83.

6. Goulart TF, Araujo-Filho VJF, Cernea CR, Matos LL. Superficial cervical plexus blockade improves pain control after thyroidectomy: A randomized controlled trial. Clinics (Sao Paulo). 2019;74:e605.

7. Gürkan Y, Taş Z, Toker K, Solak M. Ultrasound guided bilateral cervical plexus block reduces postoperative opioid consumption following thyroid surgery. J Clin Monit Comput. 2015;29(5):579-84.

8. Hu S, Shu T, Xu S, Ju X, Wang S, Ma L. Ultrasound-guided bilateral superficial cervical plexus block enhances the quality of recovery of uremia patients with secondary hyperparathyroidism following parathyroidectomy: a randomized controlled trial. BMC Anesthesiol. 2021;21(1):228.

9. Kale S, Aggarwal S, Shastri V, Chintamani. Evaluation of the Analgesic Effect of Bilateral Superficial Cervical Plexus Block for Thyroid Surgery: A Comparison of Presurgical with Postsurgical Block. Indian J Surg. 2015;77:1196-200.

10. Kannan S, Surhonne NS, Chethan Kumar R, Kavitha B, Devika Rani D, Raghavendra Rao RS. Effects of bilateral superficial cervical plexus block on sevoflurane consumption during thyroid surgery under entropy-guided general anesthesia: A prospective randomized study. Korean Journal of Anesthesiology. 2018;71:141-8.

11. Ali Majdoub1* MO, Hela Attia1, Nouha Ben Hmida2, Nabil Driss. Ultrasound-Guided Intermediate Cervical Plexus Block effects on per and

Post-Operative Opioids Consumption during Total Thyroidectomy under General

Anesthesia. Journal of Biomedical Research and Reviews2018.

12. Mariappan R, Mehta J, Massicotte E, Nagappa M, Manninen P, Venkatraghavan L. Effect of superficial cervical plexus block on postoperative quality of recovery after anterior cervical discectomy and fusion: a randomized controlled trial. Can J Anaesth. 2015;62(8):883-90.

13. Ozgun M, Hosten T, Solak M. Effect of Bilateral Superficial Cervical Plexus Block on Postoperative Analgesic Consumption in Patients Undergoing Thyroid Surgery. Cureus. 2022;14(1):e21212.

14. Mohsen Waheb WE-SAKMNMSE-SAMH. Effect Of Ultrasound Guided Superficial Cervical Plexus Block Using Combination Of Bupivacaine And Lidocaine On Intraoperative Opioid Consumption In Tympanomastoid Operations In Adults, Randomized Controlled Study. Journal of Pharmaceutical Negative Results. 2023:326-32.

15. Yao Y, Lin C, He Q, Gao H, Jin L, Zheng X. Ultrasound-guided bilateral superficial cervical plexus blocks enhance the quality of recovery in patients undergoing thyroid cancer surgery: A randomized controlled trial. J Clin Anesth. 2020;61:109651.

16. Zeng M, Li R, Xu X, Wang D, Dong J, Li S, et al. Ultrasound-guided superficial cervical plexus block reduces opioid consumption in patients undergoing craniotomy via suboccipital retrosigmoid approach: a randomized controlled trial. Reg Anesth Pain Med. 2022.

17. Abadi A, Cohen R. Evaluation of an Enhanced Recovery After Surgery Protocol Including Parasternal Intercostal Nerve Block in Cardiac Surgery Requiring Sternotomy. Am Surg. 2021;87(10):1561-4.

18. Ahmed Z, Samad K, Ullah H. Role of intercostal nerve block in reducing postoperative pain following video-assisted thoracoscopy: A randomized controlled trial. Saudi J Anaesth. 2017;11(1):54-7.

19. Chen H, Song W, Wang W, Peng Y, Zhai C, Yao L, et al. Ultrasound-guided parasternal intercostal nerve block for postoperative analgesia in mediastinal mass resection by median sternotomy: a randomized, double-blind, placebo-controlled trial. BMC Anesthesiol. 2021;21(1):98.

20. Honey RJD, Ghiculete D, Ray AA, Pace KT. A randomized, double-blinded, placebo-controlled trial of intercostal nerve block after percutaneous nephrolithotomy. Journal of Endourology. 2013;27:415-9.

21. Hsieh MJ, Wang KC, Liu HP, Gonzalez-Rivas D, Wu CY, Liu YH, et al. Management of acute postoperative pain with continuous intercostal nerve block after single port video-assisted thoracoscopic anatomic resection. Journal of Thoracic Disease. 2016;8:3563-71.

22. Kang CM, Kim WJ, Yoon SH, Cho CB, Shim JS. Postoperative Pain Control by Intercostal Nerve Block After Augmentation Mammoplasty. Aesthetic plastic surgery. 2017;41:1031-6.

23. Kang DK, Kang MK, Woon H, Hwang YH. The feasibility of thoracoscopic-guided intercostal nerve block during uniportal video-assisted thoracoscopic lobectomy of the lung. J Minim Access Surg. 2022;18(4):567-70.

24. Kang DK, Kang MK. A pilot study of intraoperative intercostal nerve block during uniportal thoracoscopic wedge resection of the lung. Ann Thorac Med. 2022;17(3):180-3.

25. Lee CY, Robinson DA, Johnson CA, Jr., Zhang Y, Wong J, Joshi DJ, et al. A Randomized Controlled Trial of Liposomal Bupivacaine Parasternal Intercostal Block for Sternotomy. Ann Thorac Surg. 2019;107(1):128-34.

26. Lewis TC, Sureau K, Katz A, Fargnoli A, Lesko M, Rudym D, et al. Multimodal opioid-sparing pain management after lung transplantation and the impact of liposomal bupivacaine intercostal nerve block. Clin Transplant. 2022;36(1):e14512.

27. Ozkan D, Akkaya T, Karakoyunlu N, Arik E, Ergil J, Koc Z, et al. Effect of ultrasound-guided intercostal nerve block on postoperative pain after percutaneous nephrolithotomy: Prospective randomized controlled study. Anaesthesist. 2013;62:988-94.

28. Ranganathan P, Tadvi A, Jiwnani S, Karimundackal G, Pramesh C. A randomised evaluation of intercostal block as an adjunct to epidural analgesia for post-thoracotomy pain. Indian Journal of Anaesthesia. 2020;64:280-5.

29. Shah A, Rowlands M, Krishnan N, Patel A, Ott-Young A. Thoracic Intercostal Nerve Blocks Reduce Opioid Consumption and Length of Stay in Patients Undergoing Implant-Based Breast Reconstruction. Plast Reconstr Surg. 2015;136(5):584e-91e.

30. Vilvanathan S, Saravanababu MS, Sreedhar R, Gadhinglajkar SV, Dash PK, Sukesan S. Ultrasound-guided Modified Parasternal Intercostal Nerve Block: Role of Preemptive Analgesic Adjunct for Mitigating Poststernotomy Pain. Anesth Essays Res. 2020;14(2):300-4.

31. Wang Y, Cheng J, Yang L, Wang J, Liu H, Lv Z. Ropivacaine for Intercostal Nerve Block Improves Early Postoperative Cognitive Dysfunction in Patients Following Thoracotomy for Esophageal Cancer. Med Sci Monit. 2019;25:460-5.

32. Wu CF, Hsieh MJ, Liu HP, Gonzalez-Rivas D, Liu YH, Wu YC, et al. Management of post-operative pain by placement of an intraoperative intercostal catheter after single port video-assisted thoracoscopic surgery: A propensity-score matched study. Journal of Thoracic Disease. 2016;8:1087-93.

33. Xu J, Pu M, Xu X, Xiang J, Rong X. The postoperative analgesic effect of intercostal nerve block and intravenous patient-controlled analgesia on patients undergoing lung cancer surgery. Am J Transl Res. 2021;13(8):9790-5.

34. Zhan Y, Chen G, Huang J, Hou B, Liu W, Chen S. Effect of intercostal nerve block combined with general anesthesia on the stress response in patients undergoing minimally invasive mitral valve surgery. Exp Ther Med. 2017;14(4):3259-64.

35. Zhu M, Gu Y, Sun X, Liu X, Chen W, Miao C. Ultrasound-Guided Intercostal Nerve Block Following Esophagectomy for Acute Postoperative Pain Relief in the Postanesthesia Care Unit. Pain Pract. 2018;18(7):879-83.

36. Elahwal L, Elrahwan S, Elbadry AA. Ilioinguinal and Iliohypogastric Nerve Block for Acute and Chronic Pain Relief After Caesarean Section: A Randomized Controlled Trial. Anesth Pain Med. 2022;12(2):e121837.

37. Gu J, Hao C, Yan X, Xuan S. Applied analysis of ultrasound-guided ilioinguinal and iliohypogastric nerve blocks in the radical surgery of aged cervical cancer. Oncol Lett. 2017;13(3):1637-40.

38. Naghshineh E, Shiari S, Jabalameli M. Preventive effect of ilioinguinal nerve block on postoperative pain after cesarean section. Adv Biomed Res. 2015;4:229.

39. Raoofi Z, Karimi MB, Parvar SP. Ilioinguinal and iliohypogastric nerve block with anatomical landmark and direct visualization technique. Bangladesh Journal of Obstetrics and Gynecology. 2014;29:83-6.
